# Supersulfide controls intestinal inflammation by suppressing CD4^+^ T cell proliferation

**DOI:** 10.3389/fimmu.2025.1506580

**Published:** 2025-04-15

**Authors:** Shunichi Tayama, Yuya Kitamura, Kyoga Hiraide, Hibiki Suzuki, Jing Li, Ziying Yang, Ryoji Mitsuwaka, Akihisa Kawajiri, Kosuke Sato, Feng Gao, Taku Nakai, Yuko Okuyama, Tadahisa Numakura, Mitsuhiro Yamada, Tomoaki Ida, Masanobu Morita, Takeshi Kawabe, Takaaki Akaike, Naoto Ishii

**Affiliations:** ^1^ Department of Microbiology and Immunology, Tohoku University Graduate School of Medicine, Sendai, Japan; ^2^ Applied Oxygen Physiology Project, New Industry Creation Hatchery Center, Tohoku University, Sendai, Japan; ^3^ Department of Oxygen Biology, Tohoku University Graduate School of Medicine, Sendai, Japan; ^4^ Department of Respiratory Medicine, Tohoku University Graduate School of Medicine, Sendai, Japan; ^5^ Organization for Research Promotion, Osaka Metropolitan University, Sakai, Japan; ^6^ Department of Environmental Medicine and Molecular Toxicology, Tohoku University Graduate School of Medicine, Sendai, Japan

**Keywords:** inflammatory bowel disease, Cd4 + t cell, cell proliferation, cell cycle, supersulfide metabolism

## Abstract

Inflammatory bowel disease (IBD) is characterized by chronic intestinal inflammation where CD4^+^ T lymphocytes play an essential role. Accumulating evidence suggests that immune responses driven by CD4^+^ T cells are critically regulated by various metabolic pathways including oxidative phosphorylation and glycolysis. Here we show that CARS2/CPERS-dependent supersulfide metabolism restrains CD4^+^ T cell proliferation in a cell-intrinsic manner. Under steady state, *Cars2* ^+/-^ mice exhibited spontaneous accumulation of effector/memory CD4^+^ T cells in the colon with age. In lymphopenic conditions, *Cars2* ^+/-^ CD4^+^ T cells showed enhanced cell cycle entry with reduced expression of a cell cycle inhibitor *Trp53* and triggered an exacerbated form of colitis, the response being rescued by treatment with a supersulfide donor glutathione trisulfide (GSSSG). Furthermore, re-analysis of publicly available gene datasets of human colonic CD4^+^ T lymphocytes revealed that downregulation of *CARS2* was associated with pathogenesis of IBD, and indeed, addition of GSSSG inhibited human CD4^+^ T cell proliferation *in vitro*. Together these observations reveal that CARS2/CPERS-dependent supersulfide metabolism is essential for homeostasis of intestinal effector/memory CD4^+^ T cells, and further suggest that dysregulation of the same metabolic pathway can lead to development of gut inflammation both in mice and humans.

## Introduction

Inflammatory bowel diseases (IBDs) consist of Crohn’s disease (CD) and ulcerative colitis and are characterized by chronic intestinal inflammation with relapse and remission. The etiology of IBD includes dysregulated immune responses against the gastrointestinal tract that can be induced by various factors such as genetic susceptibility and changes in composition of commensal flora (1). It is well known that effector CD4^+^ T cells including Th1 and Th17 subsets contribute to development and/or exacerbation of IBD (2). Indeed, treatment with antibodies against tumor necrosis factor, one of Th1-associated cytokines, has been established as the most effective therapeutic approach for IBD. Nonetheless, up to 40% of patients treated with the antibodies do not respond, and some patients who initially showed responsiveness gradually acquire resistance to the same treatment (3, 4). Antibodies against other Th1 and Th17 cytokines including IL-12/23, IFN-γ, IL-6, and IL-17A have been also tested in clinical trials, with no promising outcomes obtained (5). It is thus essential to better characterize the mechanisms of IBD pathogenesis through CD4^+^ T cell activation to develop novel therapeutic strategies.

Accumulating evidence suggests that several metabolic pathways govern homeostasis and activation of CD4^+^ T lymphocytes (6). For example, naïve CD4^+^ T cells adopt a quiescent state that requires low amounts of energy, and because of this reason, they mainly rely on mitochondrial oxidative phosphorylation for their peripheral maintenance (7). By contrast, upon antigen stimulation naïve cells are activated to reprogram their cellular metabolism to higher glycolysis to meet acute energy requirements (8). Subsequently, a small fraction of memory cells are generated, which in turn depend on mitochondrial fatty acid metabolism for their survival (9). In the context of intestinal inflammation, high glucose intake has been reported to induce overactivation of CD4^+^ T cells and exaggeration of colitis (10).

In addition to the above “conventional” metabolic pathways, our group has previously reported that sulfur metabolism regulated by mitochondrial cysteinyl-tRNA synthetase (CARS2) plays important roles in several biological responses. Specifically, CARS2 acts as a primary cysteine persulfide synthase (CPERS) and generates a highly reactive sulfur metabolite supersulfide, which contains catenated sulfur atoms (RSS_n_R; n > 1, R = hydrogen, or alkyl) and contributes to mitochondrial bioenergetics and protein persulfidation (11). Importantly, we previously reported that supersulfide has immune-suppressive function in murine macrophages and that treatment with an endogenous donor of the same metabolite protects mice from lethal endotoxin shock through inhibition of macrophage overactivation (12, 13). We further demonstrated that *Cars2* ^+/-^ mice exhibit severe symptoms of chronic obstructive pulmonary disease (COPD) (14). In consistent, a human clinical study detected lower amounts of supersulfide and CARS2 with upregulated levels of inflammatory cytokines in bronchial epithelial cells isolated from COPD patients (14, 15). These findings suggest that CARS2/CPERS-dependent supersulfide metabolism has a potential to inhibit inflammation exerted by various types of immune as well as non-immune cells. However, it remains to be determined whether CARS2/CPERS-dependent supersulfide metabolism is functional in colonic CD4^+^ T lymphocytes, and if so, how the same pathway controls their activation or proliferation especially in the context of intestinal inflammation.

In this study, we have examined the role for CARS2/CPERS-dependent supersulfide metabolism in CD4^+^ T lymphocytes both at homeostasis and in inflammatory conditions. Our observations reveal immunoregulatory function of the same metabolic pathway in murine and human CD4^+^ T cells.

## Results

### Effector/memory CD4^+^ T lymphocytes spontaneously accumulate in the large intestine of aged Cars2^+/-^ mice

In wild-type (WT) naïve CD4^+^ T cells, *Cars2* expression is downregulated after TCR stimulation *in vitro*, suggesting that CARS2/CPERS may play a role in T cell function (**Supplementary Figure S1**). To address the question of whether CARS2/CPERS-dependent supersulfide metabolism is functional in CD4^+^ T lymphocytes, we first examined thymic T cell development in WT versus *Cars2* ^+/-^ mice, the latter of which we previously reported that the same metabolic pathway is significantly reduced in (11). To do so we compared double negative (DN; CD4^-^ CD8^-^), double positive (DP; CD4^+^ CD8^+^), CD4 and CD8 single positive (SP; CD4^+^ CD8^-^ and D4^-^ CD8^+^, respectively), and Foxp3^+^ thymocytes between WT and *Cars2* ^+/-^ mice at the age of 2-3 months (young) and 12 months (old). WT and *Cars2* ^+/-^ mice exhibited the unaltered numbers of these thymocyte subsets (**Supplementary Figures S2A, B**), suggesting that CARS2/CPERS-dependent supersulfide metabolism does not significantly affect T cell development in the thymus.

**Figure 1:**
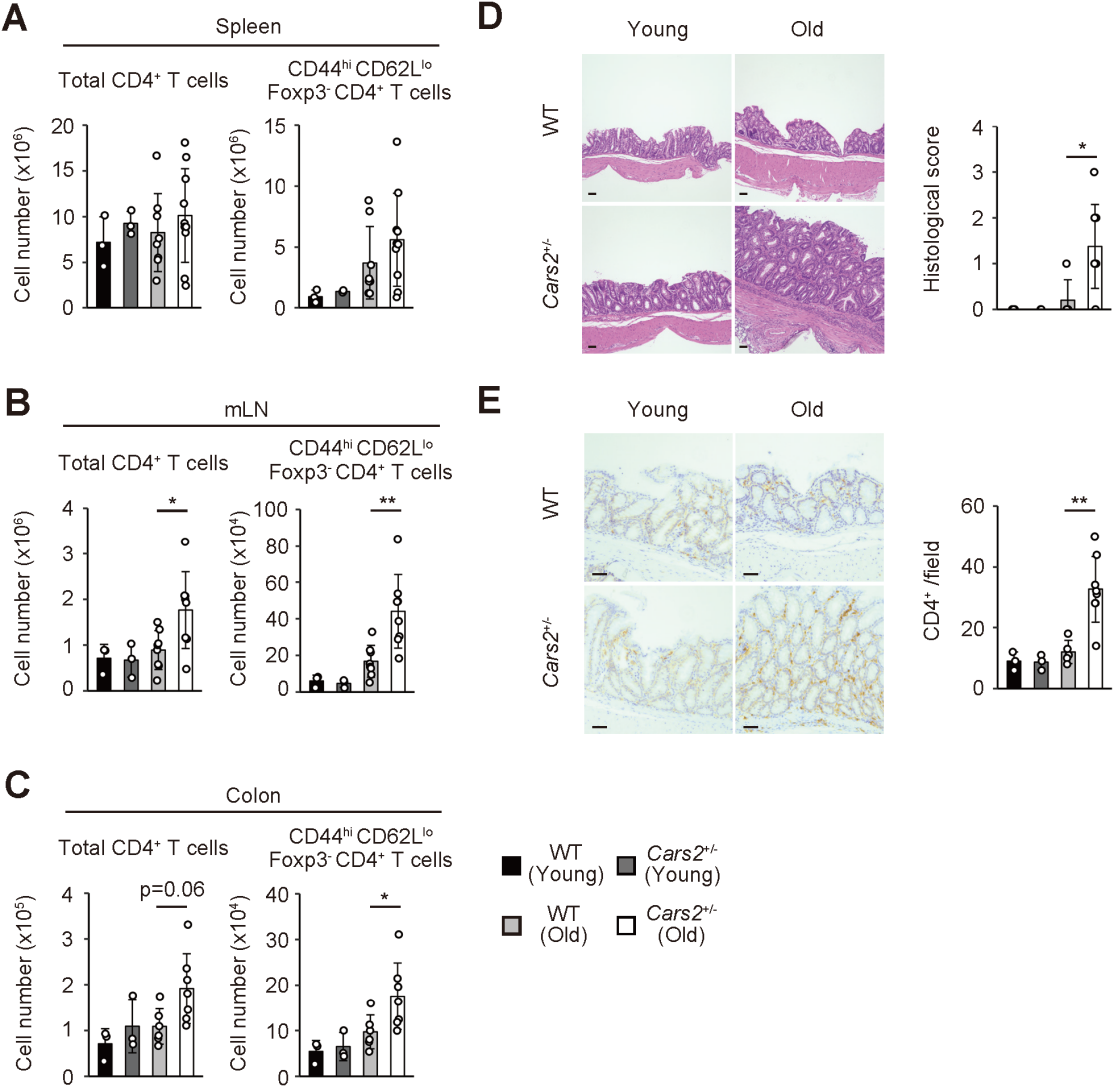
Aged *Cars2* ^+/-^ mice exhibit spontaneous accumulation of effector/memory CD4^+^ T lymphocytes in the colon. **(A-C)** Old *Cars2* ^+/-^ mice have increased number of effector/memory CD4^+^ T lymphocytes in the colon and gut-associated lymphoid tissues. The bar graphs indicate the absolute cell number of total CD4^+^ as well as CD44^hi^ CD62L^lo^ Foxp3^-^ CD4^+^ T cells in the **(A)** spleen, **(B)** mLNs, and **(C)** colon of WT and *Cars2* ^+/-^ mice at the age of 2-3 months (young) and 12 months (old) (n = 3 to 10). **(D, E)** *Cars2* ^+/-^ mice spontaneously exhibit hyperplastic mucosa with CD4^+^ T lymphocyte infiltrates in the intestine with age. The representative microscopic images display **(D)** H&E and **(E)** CD4-directed immunohistochemical staining of the colonic sections from the indicated groups while the bar graphs indicate **(D)** histological scores and **(E)** quantification of CD4-positive cells (n = 3 to 8). Data shown are pooled from two independent experiments. The data are shown as the mean ± standard deviation. Scale bars, 50 μm. *p < 0.05, **p < 0.01.

To examine if CARS2/CPERS affects peripheral CD4^+^ T cell homeostasis, we analyzed the same cells in the spleen, mesenteric lymph nodes (mLNs), and the colon obtained from young and old WT versus *Cars2* ^+/-^ mice. While CD4^+^ T cells were equally present in the spleen of WT and *Cars2* ^+/-^ mice, those and especially their CD44^hi^ CD62L^lo^ effector/memory subset in mLNs and the colon significantly increased in number in old but not young *Cars2* ^+/-^ mice (**Figures 1A–C**). In consistent, CD4^+^ T lymphocytes histologically accumulated in the colon of old *Cars2* ^+/-^ mice, which led to hyperplastic mucosa with increased inflammatory infiltrates in the same tissues (**Figures 1D, E**). Among other immune cells infiltrating in the colon, CD44^lo^ CD62L^hi^ naïve CD4^+^ T cells accumulated slightly more in the *Cars2* ^+/-^ than WT old mice, whereas we could not detect any difference in other cell types such as Foxp3^+^ Tregs, CD8^+^ T cells and neutrophils (**Supplementary Figures S3A–C**). Furthermore, the weight of each organ, colon length and body weight were not altered between WT and *Cars2* ^+/-^ old mice (**Supplementary Figures S4A–C**), and we could not detect any difference in *Cars2* expression in CD4^+^ T cells isolated from spleen and mLN between young and old WT mice (**Supplementary Figure S5**). Together these data indicate that CARS2/CPERS tonically inhibits accumulation of effector/memory CD4^+^ T cells in the colon and gut-associated lymphoid tissues at homeostasis.

### CARS2/CPERS inhibits homeostatic proliferation of CD4^+^ T cells in the gut in a cell-intrinsic manner

**Figure 2:**
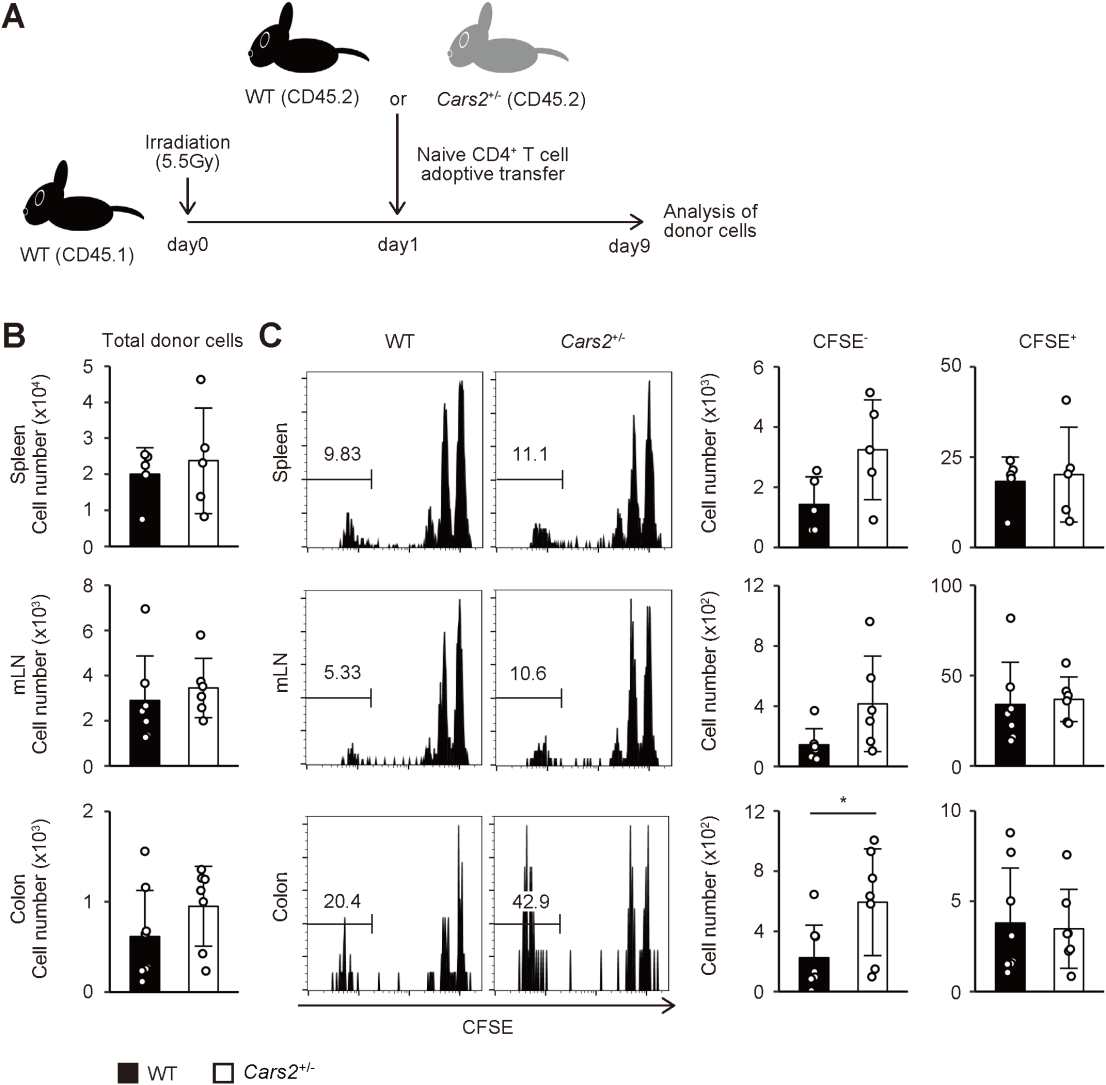
*Cars2* ^+/-^ CD4^+^ T cells exhibit robust homeostatic proliferation in acutely lymphopenic environment. **(A)** An experimental design. CFSE-labelled naïve CD4^+^ T cells from WT or *Cars2* ^+/-^ (both CD45.2) mice were transferred into sublethally irradiated (5.5 Gy) CD45.1 recipient mice, and the donor cells analyzed 9 days later. **(B)** WT and *Cars2* ^+/-^ donor cells equally dwell in the recipient mice. The bar graphs show the absolute number of donor cells in the indicated organs (n = 5 to 8). **(C)** Fast homeostatic proliferation in the gut is accelerated in *Cars2* ^+/-^ donor cells. The representative histograms depict CFSE dilution of the donor cells accumulating in the indicated organs whereas the bar graphs show the number of CFSE^-^ (> 6 divisions) and CFSE^+^ (0 - 2 divisions) cells among the donor cell populations (n = 5 to 8). Data are pooled from three independent experiments. The data are shown as the mean ± standard deviation. *p < 0.05.

Homeostasis of naïve and effector/memory CD4^+^ T lymphocytes is governed by a proliferative response called “homeostatic proliferation” (16). Experimentally, this response can be best examined in lymphopenic settings. Thus, when transferred into lymphopenic animals such as gene-manipulated or sublethally irradiated mice, some naïve CD4^+^ T cells rapidly proliferate to give rise to a subpopulation with an effector/memory phenotype in lymphoid as well as non-lymphoid tissues (17–19). To determine whether the increased number of effector/memory CD4^+^ T lymphocytes in mLNs and the colon of aged *Cars2* ^+/-^ mice (**Figures 1B, C**) is attributed to enhanced homeostatic proliferation, we next sought to examine the same proliferative response of naïve CD4^+^ T cells derived from WT versus *Cars2* ^+/-^ animals. To do so we isolated naïve cells from these two types of mice, labeled with carboxyfluorescein diacetate succinimydyl ester (CFSE), transferred into congenic recipients rendered acutely lymphopenic by sublethal irradiation, and analyzed the donor cells in the spleen, mLNs, and the colon 9 days later (the experimental design shown in **Figure 2A**). While the total donor cell number was unaltered between WT and *Cars2* ^+/-^ groups (**Figure 2B**), *Cars2* ^+/-^ as compared to WT CD4^+^ T cells more rapidly proliferated in the colon as reflected by the heightened CFSE^-^ fractions (CD44^hi^ CD62L^lo^), with the slowly proliferating CFSE^+^ cells (CD44^lo^ CD62L^hi^) largely unchanged (**Figure 2C**, **Supplementary Figure S6**). Thus, CARS2/CPERS suppresses fast but not slow homeostatic proliferation of naïve CD4^+^ T lymphocytes in a cell-intrinsic manner.

### CARS2/CPERS suppresses proliferation of colitogenic CD4^+^ T cells

**Figure 3:**
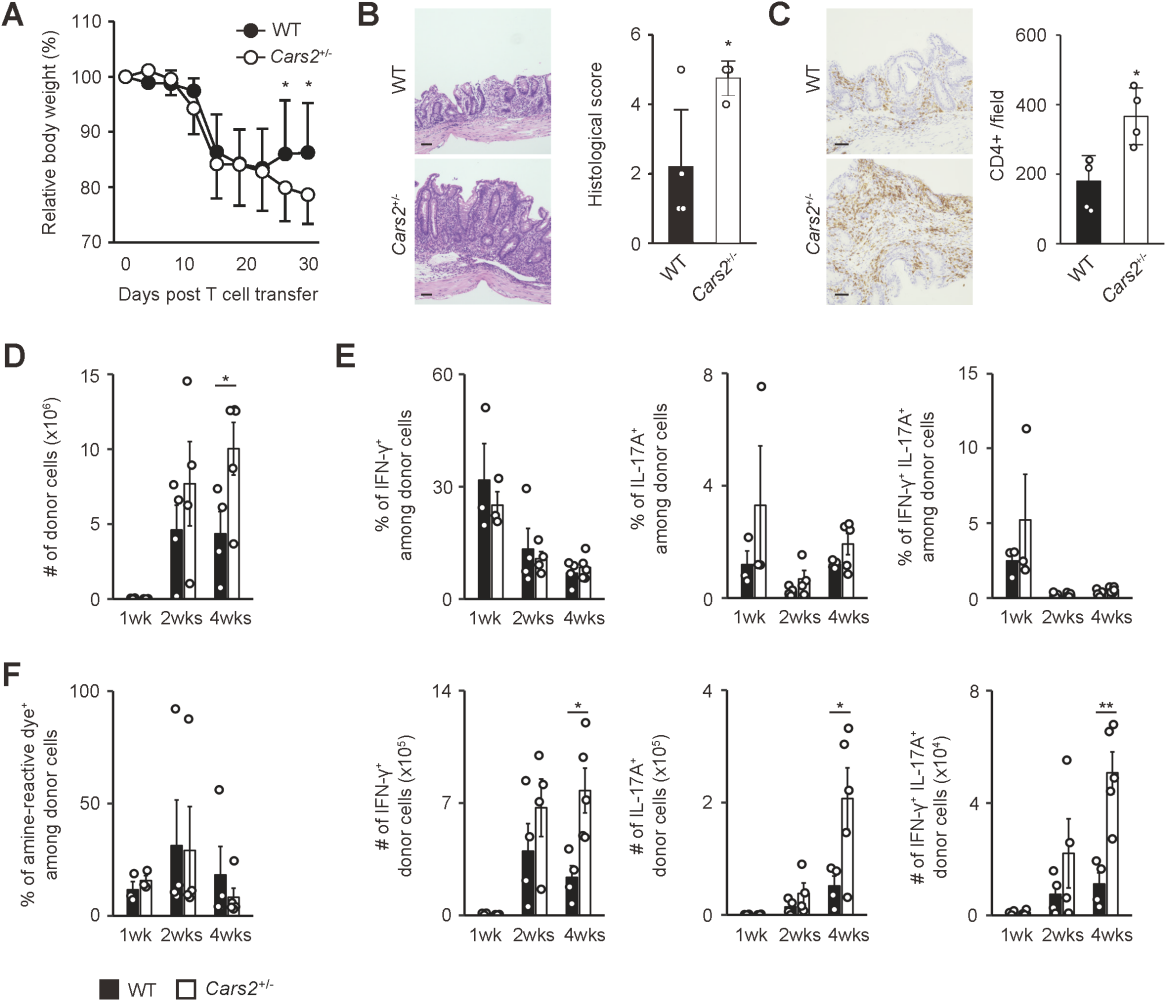
*Cars2* ^+/-^ CD4^+^ T cells trigger exacerbated colitis in *Rag2* ^-/-^ mice. **(A)** *Cars2* ^+/-^ versus WT naïve CD4^+^ T lymphocytes induce exaggerated body weight loss in *Rag2* ^-/-^ mice. Naïve CD4^+^ T cells derived from WT or *Cars2* ^+/-^ mice were transferred into *Rag2* ^-/-^ animals, and the hosts analyzed at different time points. The graph shows relative body weight of the recipient mice (n = 7 to 8). **(B, C)** *Cars2* ^+/-^ CD4^+^ T cells trigger severe histological colitis. Donor cells were transferred into *Rag2* ^-/-^ mice as described above, and the hosts analyzed 4 weeks later. The representative microscopic images of colonic sections display **(B)** H&E and **(C)** CD4-directed immunohistochemical staining while the bar graphs indicate **(B)** the histological scores as well as **(C)** quantification of CD4-positive cells in each group (n = 4 to 5). **(D)** *Cars2* ^+/-^ CD4^+^ T lymphocytes accumulate in the intestine to a greater degree than do WT controls. The bar graph shows the absolute number of intestinal donor cells (CD3^+^ CD4^+^ Foxp3^-^ CD44^hi^ CD62L^lo^) at the indicated time points (n = 3 to 5). **(E)** Th1 as well as Th17 differentiation rate is essentially the same between WT and *Cars2* ^+/-^ donor cells. Several weeks after transfer into *Rag2* ^-/-^ mice, donor cells were measured for IFN-γ as well as IL-17A expression. Bar graphs indicating (top) the frequency and (bottom) the absolute number of cytokine-producing cells among the total donor population accumulating in the colon are depicted (n = 3 to 5). **(F)** The rate of cell death is comparable between WT and *Cars2* ^+/-^ donor cells. In the above experiments donor cells were stained with amine-reactive dye. The bar graph indicates the frequency of amine-reactive dye^+^ (dead) cells among total donor population in the colon (n = 3 to 5). Data are **(A, D-F)** pooled from and **(B, C)** representative of two independent experiments performed. The data are shown as the mean ± standard deviation. Scale bars, 50 μm. *p < 0.05, **p < 0.01.

Based on previous findings demonstrating a correlation between the degree of homeostatic proliferation of CD4^+^ T cells and severity of colitis (20, 21), we hypothesized that enhanced homeostatic proliferation of *Cars2* ^+/-^ naive CD4^+^ T cells can exacerbate colitis in certain circumstances. To seek this possibility, we used a mouse model of colitis where naïve CD4^+^ T cells are transferred into lymphodeficient mice (21–23). In such chronically lymphopenic environment naive CD4^+^ T cells robustly proliferate to trigger colitis through fast homeostatic proliferation (16). Using this approach, we adoptively transferred WT or *Cars2* ^+/-^ naïve CD4^+^ T cells into *Rag2* ^-/-^ mice and analyzed the donor cells as well as the host animals at different time points. *Rag2* ^-/-^ hosts that received *Cars2* ^+/-^ versus WT CD4^+^ T cells exhibited severer body weight loss and histological colitis (**Figures 3A–C**). In consistent, the number of *Cars2* ^+/-^ as compared to WT donor cells was significantly higher in the gut but not in lymphoid organs 4 weeks after transfer (**Figure 3D** and **Supplementary Figures S7A, D**). Furthermore, the frequency of effector cytokine-producing cells (IFN-γ^+^, IL-17A^+^, and IFN-γ^+^ IL-17A^+^) among the total donor population was comparable between the two groups in all of the organs examined, and as a consequence, the total number of cytokine-secreting donor cells accumulating in the gut 4 weeks after transfer was higher in the *Cars2* ^+/-^ CD4^+^ T cell-transferred group (**Figure 3E**, **Supplementary Figures S7B, E**). Because the frequency of dead cells was equivalent between the two groups (**Figure 3F**, **Supplementary Figures S7C, F**) and because the *in vitro* assay demonstrated that CARS2 deficiency had no effect on T cell activation and differentiation (**Supplementary Figures S8A, B**), these results suggest that augmented accumulation of cytokine-secreting *Cars2* ^+/-^ CD4^+^ T cells in the colon may reflect an enhanced rate of lymphopenia-induced homeostatic proliferation.

**Figure 4:**
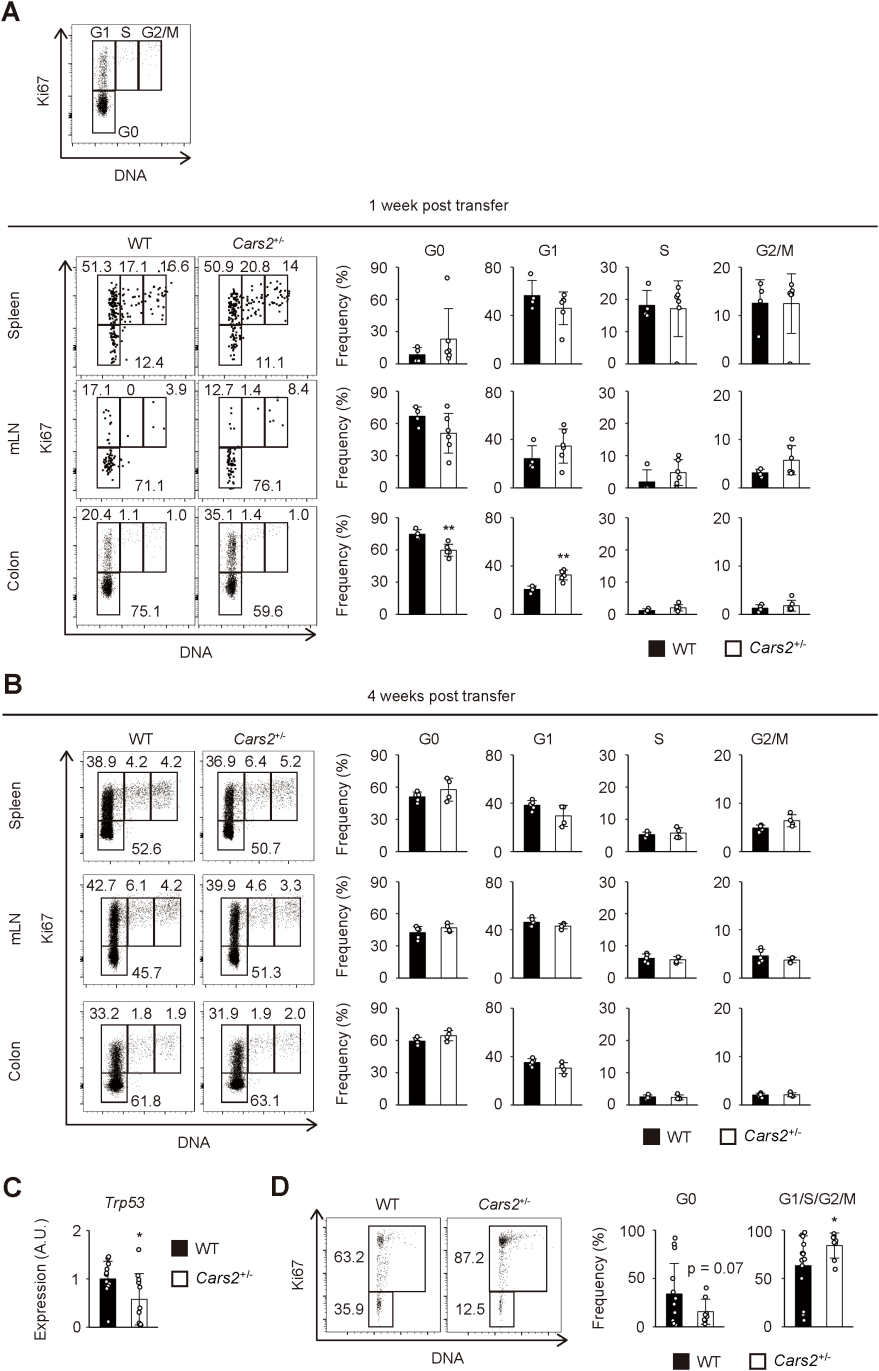
Cell cycle entry is accelerated in *Cars2* ^+/-^ CD4^+^ T cells at an early phase of colitis. **(A, B)** *Cars2* ^+/-^ CD4^+^ T cells more efficiently enter the cell cycle than do WT controls at an early stage of colitis. Naïve CD4^+^ T cells derived from WT or *Cars2* ^+/-^ animals were transferred to *Rag2* ^-/-^ host mice as described in **Figure 3**, and the donor cells analyzed at the indicated time points. The representative dot plots display expression levels of Ki67 and total amounts of DNA in donor cells while the bar graphs show the frequency of cells within the indicated cell cycle phases among total donor populations at **(A)** 1 week and **(B)** 4 weeks post transfer (n = 4 to 6). A representative dot plot depicting gating strategies for G0, G1, S, and G2/M cells is also included. **(C)** Colonic *Cars2* ^+/-^ T cells express lower levels of *Trp53*. Real-time PCR was performed to detect *Trp53* expression. The bar graph shows relative expression of *Trp53* in donor cells (n = 11 to 12). **(D)** *Cars2* ^+/-^ CD4^+^ T lymphocytes enter cell cycle more efficiently *in vitro*. *Cars2* ^+/-^ naïve CD4^+^ T cells were stimulated with CD3 and CD28, and cell cycle phases analyzed 3 days later. The representative dot plots show the expression levels of Ki67 and the total amounts of DNA in CD4^+^ T cells, while the bar graphs show the frequency of cells within the indicated cell cycle phases (n = 8 to 13). Data shown are **(A, B)** representative of two independent experiments and pooled from **(D)** three and **(C)** four independent experiments. The data are shown as the mean ± standard deviation. *p < 0.05, **p < 0.01.

To test this hypothesis, we performed cell cycle analysis of the donor cells during the course of the experiments described above. One week after naïve CD4^+^ T cell transfer, the frequency of cells in G0 phase was significantly lower in *Cars2* ^+/-^ versus WT donor cells while that of cells in G1 phase higher in the former donor cell population, with cells in S and G2/M phases largely unaffected (**Figure 4A**). These differences were exclusively detected in donor cells accumulating in the gut but not in lymphoid organs (**Figure 4A**), and were not observed at a later time point (**Figure 4B**). In consistent, we detected downregulation of a cell cycle inhibitor *Trp53* in *Cars2* ^+/-^ CD4^+^ T cells at one week post transfer (**Figure 4C**). Furthermore, when naive CD4^+^ T cells were cultured *in vitro* in the presence of CD3/CD28 antibodies, *Cars2* ^+/-^ CD4^+^ T cells exhibited a higher frequency of cell cycle entry (**Figure 4D**). Together these data demonstrate that CARS2/CPERS suppresses cell cycle entry of pathogenic CD4^+^ T cells in a cell-intrinsic manner at an early stage of intestinal inflammation.

### Cars2^+/-^ regulatory T cells show unaltered differentiation and suppressive function in colitis

Naïve CD4^+^ T cells generate a few Foxp3^+^ regulatory T cells (Tregs) when transferred into lymphopenic mice (24). To examine whether CARS2/CPERS affects differentiation and/or suppressive function of the latter cells, we analyzed Tregs that differentiate from naïve CD4^+^ T cells in the above colitis model. To do so we transferred naïve CD4^+^ T lymphocytes into *Rag2* ^-/-^ animals and analyzed the donor cells at different time points. As shown in **Supplementary Figure S9A**, there was no difference in the number of generated Tregs, making the possibility unlikely that CARS2/CPERS is essential for differentiation of Tregs from naïve precursors. To further determine whether CARS2/CPERS affects suppressive function of Tregs, we co-transferred *Cars2* ^+/+^ Foxp3^-^ naïve CD4^+^ T cells together with Foxp3^+^ cells of *Cars2* ^+/+^ or *Cars2* ^+/-^ origins into *Rag2* ^-/-^ mice. The two types of Tregs equally suppressed naive cell-induced colitis (**Supplementary Figure S9B**). Hence, CARS2/CPERS appears to be dispensable for Treg differentiation and function.

### GSSSG treatment ameliorates colitis triggered by Cars2^+/-^ naïve CD4^+^ T cells

**Figure 5:**
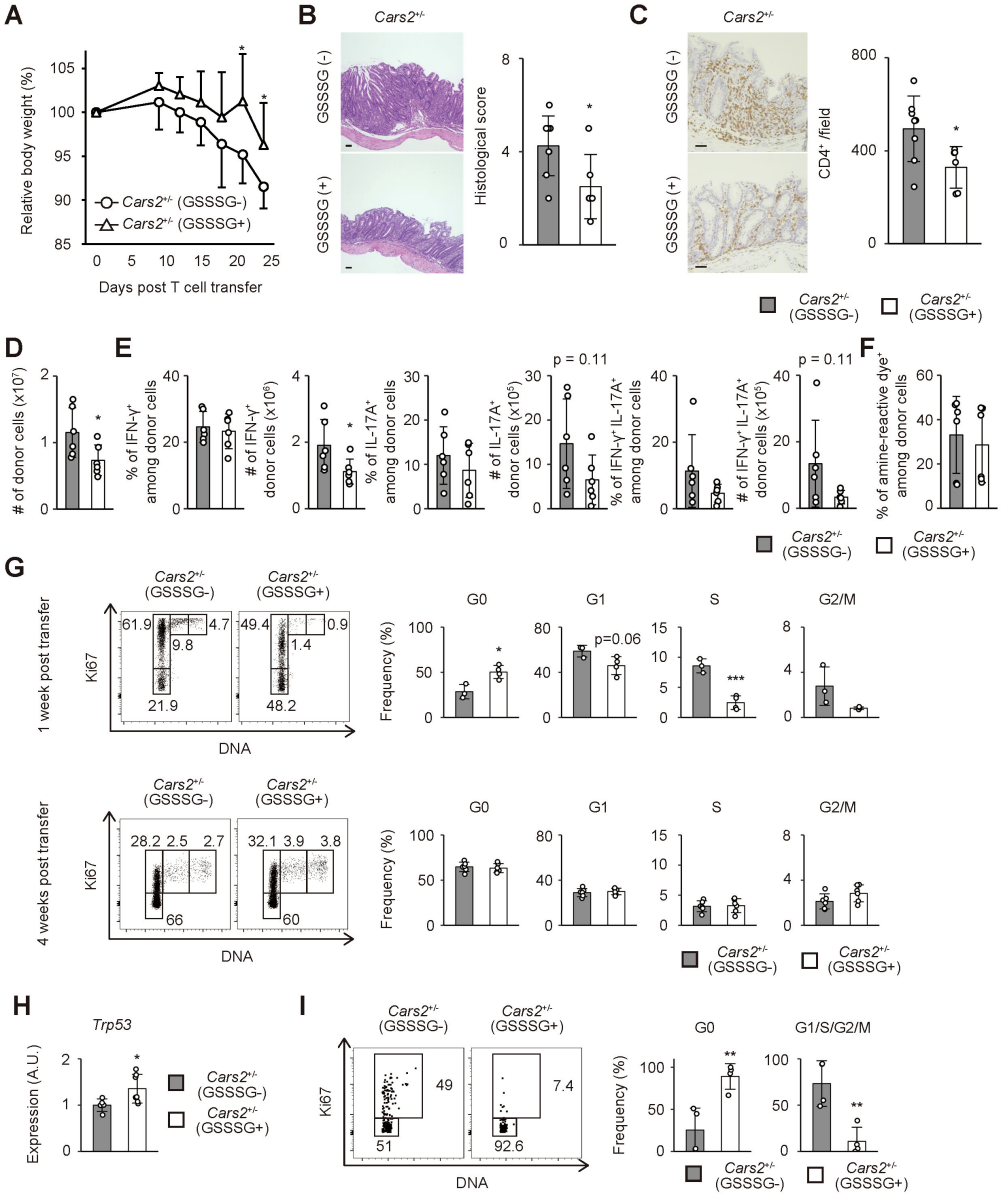
Treatment with GSSSG ameliorates colitis in *Rag2* ^-/-^ mice that received *Cars2* ^+/-^ CD4^+^ T cells. **(A)** GSSSG treatment inhibits body weight reduction of *Rag2* ^-/-^ mice that have received *Cars2* ^+/- ^naïve CD4^+^ T lymphocytes. *Cars2* ^+/-^ naïve CD4^+^ T cells were transferred into *Rag2* ^-/-^ hosts that were then daily treated with phosphate-buffered saline (PBS) or GSSSG. The graph shows relative body weight of the recipient mice at the indicated time points (n = 6 to 8). **(B, C)** Histological colitis triggered by *Cars2* ^+/-^ naïve CD4^+^ T cells is ameliorated by supplementation with GSSSG. Representative microscopic images of colonic sections showing **(B)** H&E and **(C)** CD4-directed immunohistochemical staining together with bar graphs indicating **(B)** histological scores as well as **(C)** quantification of CD4-positive cells are displayed (n = 6 to 8). **(D)** GSSSG treatment inhibits accumulation of *Cars2* ^+/-^ CD4^+^ T lymphocytes in the colon. The bar graph shows the absolute number of intestinal donor cells at 1 month post transfer (n = 6). **(E)** GSSSG inoculation does not affect differentiation of Th1 or Th17 cells. Four weeks after transfer into *Rag2* ^-/-^ mice supplemented with PBS or GSSSG, donor cells were measured for IFN-γ as well as IL-17A expression. Bar graphs indicating the frequency and the absolute number of cytokine-producing cells among the total donor population accumulating in the colon are depicted (n = 6). **(F)** The rate of cell death in *Cars2* ^+/-^ donor cells are comparable between PBS and GSSSG treated groups. In the above experiments donor cells were stained with amine-reactive dye. The bar graph indicates the frequency of amine-reactive dye^+^ (dead) cells among total donor population in the colon (n = 6). **(G)** GSSSG administration inhibits cell cycle entry of *Cars2* ^+/-^ CD4^+^ T cells at an early phase of colitis. The representative dot plots show expression levels of Ki67 and total amounts of DNA in donor cells while the bar graphs show the frequency of cells within the indicated cell cycle phases among total donor populations at 1 week and 4 weeks post transfer (n = 3 to 6). **(H)** GSSSG administration upregulates *Trp53* expression in colonic *Cars2* ^+/-^ T cells. A bar graph showing relative expression of *Trp53* in donor cells at 1 week after transfer is displayed (n = 5 to 7). **(I)** GSSSG suppresses cell cycle entry of *Cars2* ^+/-^ CD4^+^ T lymphocytes *in vitro*. *Cars2* ^+/-^ naïve CD4^+^ T cells were stimulated with CD3 and CD28 in the presence or absence of GSSSG, and the cell cycle phases analyzed 3 days later. The representative dot plots show expression levels of Ki67 and total amounts of DNA in CD4^+^ T cells while the bar graphs show the frequency of cells within the indicated cell cycle phases (n = 4). Data are pooled from two or three independent experiments. The data are shown as the mean ± standard deviation. Scale bars, 50 μm. *p < 0.05, **p < 0.01, ***p < 0.001.

CARS2/CPERS regulates sulfur metabolism through the production of supersulfide (11). To ask whether CARS2/CPERS exerts its suppressive activity on colitogenic T cells via supersulfide, we administered a supersulfide donor glutathione trisulfide (GSSSG) to *Rag2* ^-/-^ mice that had received naïve CD4^+^ T lymphocytes. Treatment with GSSSG significantly ameliorated body weight reduction and histological colitis induced by *Cars2* ^+/-^ naïve CD4^+^ T cell transfer (**Figures 5A–C**). On the other hand, the same treatment did not rescue severity of colitis driven by WT CD4^+^ T cells (**Supplementary Figures S10A–C**). Further analysis of *Cars2* ^+/-^ donor cells revealed that GSSSG inoculation significantly repressed the absolute number of donor cells accumulating in the gut (**Figure 5D**). While the frequency of cytokine-producing cells among gut-infiltrating CD4^+^ T cells was unchanged regardless of GSSSG supplementation, their absolute numbers were lowered in the presence of GSSSG (**Figure 5E**). The same treatment had no influence on cell death of *Cars2* ^+/-^ CD4^+^ T cells (**Figure 5F**). These data argue that augmented severity of colitis in *Rag2* ^-/-^ mice that have received *Cars2* ^+/-^ CD4^+^ T cells is attributed to insufficient amounts of supersulfide in the same cells.

We next examined cell cycle status of *Cars2* ^+/-^ donor cells in the above experiments. As shown in **Figure 5G**, GSSSG treatment significantly suppressed cell cycle entry of CD4^+^ T cells at 1 but not 4 week(s) post transfer. Moreover, *Trp53* expression in CD4^+^ T cells was upregulated by GSSSG administration (**Figure 5H**). To further confirm whether GSSSG can directly act on CD4^+^ T cells, we stimulated *Cars2* ^+/-^ naïve CD4^+^ T cells *in vitro* and analyzed their cell cycle status in the presence or absence of GSSSG. Addition of GSSSG diminished the frequency of cycling cells while increased the quiescent fraction (**Figure 5I**). Together these findings suggest that supersulfide can inhibit cell cycle entry of CD4^+^ T cells.

### Reduced expression of CARS2 in human CD4^+^ T cells correlates with pathogenesis of IBD

**Figure 6:**
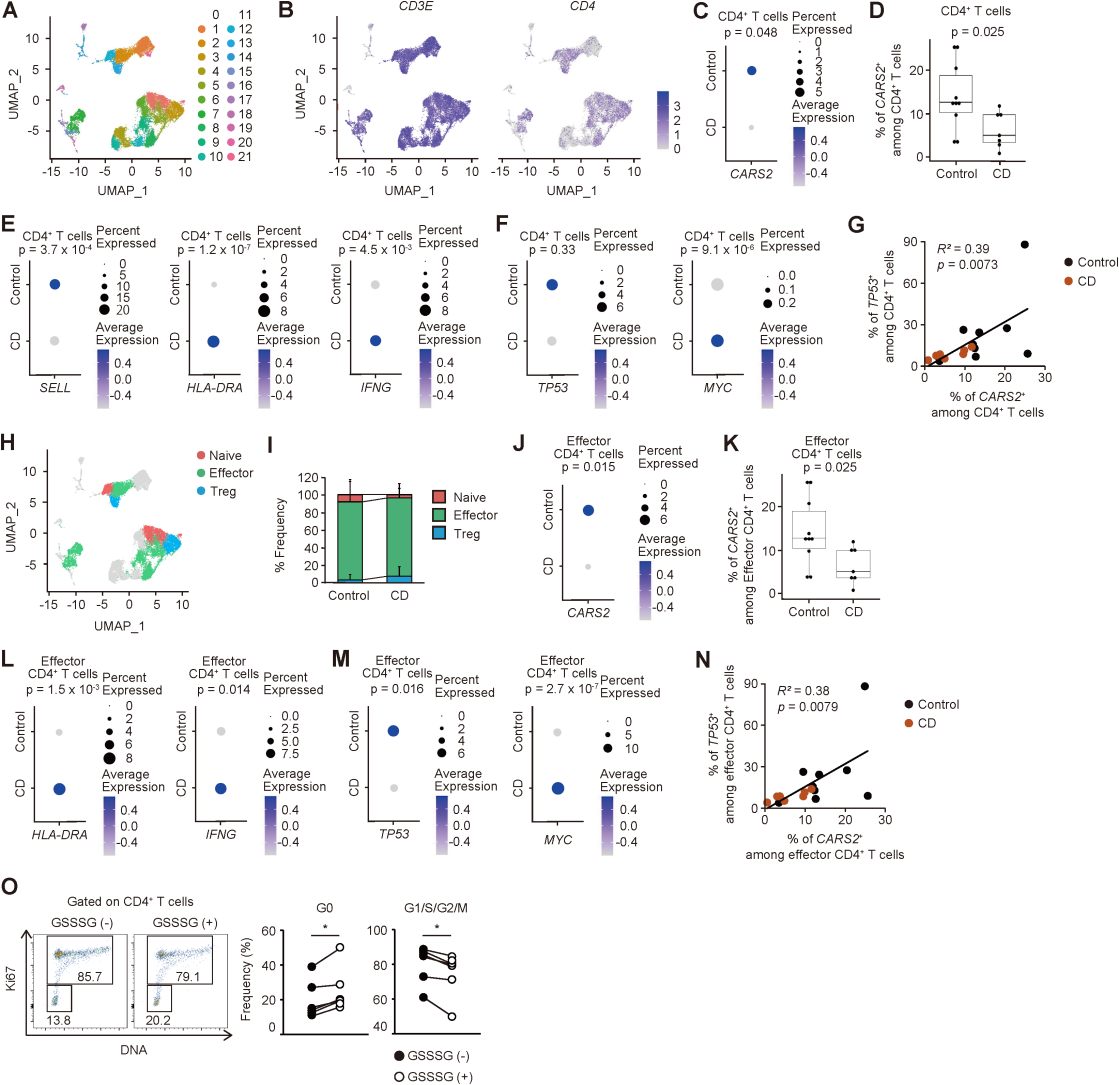
CARS2-dependent regulation of CD4^+^ T cell proliferation is operative in humans. **(A-N)** Intestinal CD4^+^ T cells derived from CD patients exhibit lower expression of *CARS2* with highly proliferative signatures. Publicly available gene expression datasets of gut-infiltrating T lymphocytes from CD patients and controls were re-analyzed as described in Materials and Methods. **(A)** The plot displays re-clustered, *CD3E*-expressing cells, determined by the Uniform Manifold Approximation and Projection (UMAP) algorithm. Each dot represents a cell, and colors highlight unsupervised cell clusters. **(B)** The UMAP plots represent expression of *CD3E* and *CD4*. **(C)** The dot plot shows the frequency of *CARS2*-expressing cells and the relative expression level in CD4^+^ T cells. *P* values for expression level of signature gene are shown. **(D)** The box plot indicates the frequency of *CARS2*-expressing cells among CD4^+^ T cells (control, n = 10; CD, n = 7). **(E, F)** The dot plots show the frequency of gene-expressing cells and the relative expression levels in CD4^+^ T cells. *P* value for the expression levels of signature gene is shown. **(G)** Correlation between the frequency of *CARS2*-positive cells and those of *TP53*-expressing CD4^+^ T cells (control, n = 10; CD, n = 7). **(H)** The UMAP plot displays *CD4*-expressing cells extracted from *CD3E*-expressing cells. Each dot represents a cell, and colors highlight individual cell subsets. **(I)** The stacked bar graph shows the proportion of the indicated cell types. **(J)** The dot plot shows the frequency of *CARS2*-expressing cells and the relative expression level in effector CD4^+^ T cells. *P* value for the expression level of the signature gene is shown. **(K)** The box plot indicates the frequency of *CARS2*-expressing cells among effector CD4^+^ T cells (control, n = 10; CD, n = 7). **(L, M)** Dot plots show the frequency of gene-expressing cells and the relative expression levels in effector CD4^+^ T cells. *P* values for expression levels of signature genes are shown. **(N)** Correlation between the frequency of *CARS2*-positive cells and that of *TP53*-expressing effector CD4^+^ T cells (control, n = 10; CD, n = 7). **(O)** GSSSG inhibits cell cycle entry of activated CD4^+^ T lymphocytes in humans. Human naïve CD4^+^ T cells were stimulated with CD3 and CD28 in the presence or absence of GSSSG, and cell cycle phases analyzed 48 hours later. The representative dot plots display expression levels of Ki67 and total amounts of DNA while the graphs show the frequency of cells within the indicated cell cycle phases among CD4^+^ T lymphocytes (n = 6). Data presented in **(O)** are pooled from three independent experiments. The data are shown as mean ± standard deviation. *R^2^*, Pearson’s correlation coefficient. *p < 0.05.

The forementioned results show that reduced levels of CARS2/CPERS-dependent supersulfide metabolism lead to excess proliferation of CD4^+^ T lymphocytes, thereby contributing to intestinal inflammation in mice. To gain insight into the question of whether lowered levels of *CARS2* in CD4^+^ T cells are associated with pathogenesis of IBD in humans, we re-analyzed publicly available datasets of single cell RNA sequencing of intestinal lamina propria mononuclear cells obtained from CD patients versus controls (25, 26). After combining samples from 10 CD patients and 7 controls, we annotated T cell clusters based on the unique signature gene *CD3E* (**Supplementary Figures S11A, B**). We next performed re-clustering of the *CD3E*-positive T cell fraction and identified 22 subclusters (**Figure 6A**, **Supplementary Figure S11C**). Among them, clusters 0, 2, 3, 5, 7 – 9, 12, 13, 15, 21 expressed both *CD3E* and *CD4*, and defined them as CD4^+^ T cells (**Figures 6A, B**, **Supplementary Figure S11C**), while cluster 1, 4, 6, 10, 11, 15, 16, 19 – 21 were CD8^+^ T cells expressing *CD8A*. Intriguingly, expression levels of *CARS2* were lower in CD4^+^ T lymphocytes in CD patients as compared to controls (**Figure 6C**). Additionally, the frequency of *CARS2*-expressing cells was lower in CD4^+^ T cells in the former group (**Figure 6D**). These findings suggest that CARS2/CPERS may be functional in human CD4^+^ T cells in the development and/or augmentation of IBD.

To further characterize the profiles of CD4^+^ T cells, we compared naive and effector markers between CD patients and controls. CD4^+^ T cells from CD patients exhibited lower levels of a naïve marker *SELL* and higher levels of activation markers *HLA-DRA* and *IFNG* (27) (**Figure 6E**). Similarly, expression of the proliferation marker *MYC* was high in CD4^+^ T cells of CD origins (28) (**Figure 6F**). While the expression level of *TP53* was not significantly different in CD4^+^ T cells, the frequency of *CARS2*-positive cells among CD4^+^ cells positively correlated with that of *TP53*-positive cells (**Figures 6F, G**). To further clarify the profiles of each gene in CD4^+^ T cells, we subclustered the same cells based on gene expression patterns (**Supplementary Figure S11C**). Within CD4^+^ T cells, cluster 0, 12, 21 showed the marker of naïve CD4^+^ T cells: *SELL*, *LEF1* and *CCR7* (29). Cluster 2, 3, 7, 8, 9, 15 corresponded to effector CD4^+^ T cells expressing *TIMP1*, *LGALS1*, *STAT1*, *SOCS1*, *ODC1*, and/or *IL2* (29, 30). Cluster 5 and 13 were both Treg based on *FOXP3* expression. Because approximately 90% of CD4^+^ T cells are composed of effector subset (**Figures 6H, I**), we focused on the effector subset in the later analysis. Similar to the total CD4^+^ T cells, *CARS2* expression and frequency were both lower in effector CD4^+^ T cells in CD patients (**Figures 6J, K**). Furthermore, effector CD4^+^ T cells from CD patients exhibited higher levels of activation markers (*HLA-DRA*, *IFN-G*) and proliferation marker *MYC* while that of cell cycle inhibitor *TP53* was lower (**Figures 6L, M**). Moreover, the frequency of *CARS2*-expressing cells among effector CD4^+^ cells positively correlated with that of *TP53*-positive cells (**Figure 6N**). These findings suggest that downregulation of CARS2/CPERS-dependent supersulfide metabolism in CD4^+^ T cells is associated with the pathogenesis of IBD in humans.

Last, we sought to formally test the relationship between CARS2-dependent supersulfide metabolism and cell cycle in human CD4^+^ T cells. To do so we isolated naïve CD4^+^ T cells from human healthy donors, stimulated with CD3/28 antibodies in the presence or absence of GSSSG, and analyzed the cultured cells 48 hours later. As shown in **Figure 6O**, addition of GSSSG significantly suppressed cell cycle entry of CD4^+^ T lymphocytes. Thus, CARS2/CPERS-dependent supersulfide metabolism inhibits CD4^+^ T cell proliferation by suppressing cell cycle entry in humans.

## Discussion

In the present study, we have shown that CARS2/CPERS-dependent supersulfide metabolism maintains homeostasis of intestinal effector/memory CD4^+^ T lymphocytes by inhibiting their excess proliferation. Indeed, CD4^+^ T cells with reduced levels of CARS2/CPERS exhibited accelerated cell cycle entry and induced severer colitis in *Rag2* ^-/-^ mice, and treatment with GSSSG, an endogenous donor of supersulfide that is generated by CARS2/CPERS, ameliorated inflammation. Importantly, this CARS2/CPERS-dependent inhibitory mechanism of CD4^+^ T cell proliferation seems to be operative also in humans, as evidenced by the findings that IBD patients had rapidly proliferating CD4^+^ T cells with lower CARS2/CPERS expression and that CD4^+^ T cell proliferation was inhibited by GSSSG supplementation *in vitro*. Together our results indicate that dysregulated CARS2/CPERS-dependent supersulfide metabolism in CD4^+^ T cells can lead to intestinal inflammation, and further suggest the same metabolic pathway as a potential therapeutic target for IBD treatment.

Supersulfide is a sulfur metabolite that critically contributes to the mitochondrial energy production and protein polysulfidation (31). In the past, supersulfide was assumed to be only generated by two enzymes cystathionine beta-synthetase (CBS) and cystathionine gamma-lyase (CSE) that both use cystine as a substrate (32). However, because the *K*m value of CBS/CSE is much higher than physiological intracellular concentration of cystine (32), it was postulated that other enzymes may play a primary role in producing supersulfide. We have previously identified CARS2/CPERS as the key enzyme that produces supersulfide by using cysteine as a substrate under physiological conditions in mammalian cells (11). Indeed, deficiency in CBS and/or CSE only partially reduces supersulfide production (33–37) whereas *Cars2* ^+/-^ cells exhibit substantial decrement of the same molecule (11, 38). Based on these findings, we proposed that CARS2/CPERS rather than CBS/CSE-dependent pathway is the primary mechanism that governs supersulfide-dependent sulfur metabolism.

In the present study, we have shown to our knowledge for the first time that CARS2/CPERS-dependent supersulfide metabolism is functional in murine colonic CD4^+^ T lymphocytes. Specifically, the same pathway inhibits intestinal CD4^+^ T cell proliferation without affecting Th1 or Th17 differentiation. Treg differentiation or function was not impaired in *Cars2* ^+/-^ CD4^+^ T cells (**Supplementary Figure S9**), which is in contrast to a previous report showing that *Cbs* ^-/-^ mice exhibit impaired Treg development and function (39). Because deficiency in CBS minimally affects supersulfide production, CBS may regulate Treg differentiation and function via the supersulfide-independent mechanism. Further investigation will be necessary to delineate relative importance of supersulfide-dependent versus -independent pathways in distinct types of CD4^+^ T cell subsets.

Our results demonstrate that CARS2/CPERS negatively regulates CD4^+^ T cell homeostatic proliferation. It is well documented that the same proliferative response is driven by T cell receptor (TCR) signaling provided by self as well as foreign antigens (16). Since we recently found that exogenous administration of GSSSG inhibits TCR signaling and protects allergen-induced airway inflammation (40), reduction of CARS2/CPERS expression in CD4^+^ T cells may enhance homeostatic proliferation via augmented TCR signaling. In addition, we observed that under inflammatory conditions, accelerated cell cycle entry of *Cars2* ^+/-^ CD4^+^ T cells was accompanied by downregulation of *Trp53*. In this regard, a previous papers pointed out that p53 acts as a cell cycle initiator in mammalian cells by enhancing and/or suppressing targets for cyclin-dependent kinase inhibitors and promoters, respectively (41, 42). Furthermore, in CD4^+^ T cells, it has been demonstrated that antigen stimulation decreases p53 expression and thereby enhances their cell cycling (43), suggesting that CARS2/CPERS-dependent supersulfide metabolism inhibits T cell proliferation through p53-mediated cell cycle inhibition.

It is noteworthy that the suppressive function of CARS2/CPERS in CD4^+^ T cell proliferation was most conspicuous in the colon. This phenomenon may be explained by the findings that colonic mucosa is enriched in commensal microbiota that can generate CARS2/CPERS substrates cysteine and its related metabolites that adopt a state of di- or tri-peptides, which are generated through the degradation of dietary and host proteins (44). Furthermore, colonic epithelial cells are known to elevate expression levels of peptide transporter 1 (PepT1), which can transport amino acid-containing peptides, under inflammatory conditions (44–47). These observations suggest that the colonic environment is rich in CARS2/CPERS substrates. It is thus possible that colonic CD4^+^ T lymphocytes may more efficiently utilize environmental cysteine than those in the other organs. In addition, CARS2/CPERS product supersulfide is also produced by commensal bacteria, which may further enhance sulfur metabolism in the intestine (48).

GSSSG administration ameliorated colitis induced by *Cars2* ^+/-^ but not WT CD4^+^ T cells (**Figure 5**, **Supplementary Figure S10**). As mentioned above, the enteric environment is a potentially good reservoir for sulfur metabolites and may be rich in GSSSG substrates. Thus, it is possible that WT T cells can synthesize sufficient amount of GSSSG by themselves and are less reactive to external exposure to the same molecule in intestinal inflammation. Furthermore, differences in environmental conditions between the *in vitro* culture system and the *in vivo* colonic environment may define the differential reactivity of healthy human CD4^+^ T cells (*in vitro*, GSSSG reactive) and WT naïve CD4^+^ T cells (*in vivo*, GSSSG non-reactive).

In summary, our present study establishes the essential role for CARS2/CPERS-dependent supersulfide metabolism in homeostasis of CD4^+^ T cells by suppressing their excess proliferation in mice. Because IBD patients had rapidly proliferating, activated CD4^+^ T lymphocytes with downregulated *CARS2* expression and because human T cell proliferation was inhibited by GSSSG treatment (**Figure 6**), our data strongly suggest that supersulfide metabolic pathway is functional in human CD4^+^ T lymphocytes as well. These observations further raise the possibility that the same metabolic pathway can be a novel therapeutic target for IBD treatment. While the current study reveals the indispensable role for the supersulfide metabolic pathway in CD4^+^ T lymphocytes, its functional significance in the other cells including intestinal epithelial as well as stromal cells remains unclear. It is thus essential to clarify its differential roles in distinct types of cells in a comprehensive manner to validate the above hypothesis in future studies.

## Materials and methods

### Mice

C57BL/6 CD45.2^+^ WT mice were purchased from Japan SLC (Hamamatsu, Japan). *Rag2* ^-/-^ and CD45.1^+^ WT mice were obtained from breeding stock at Tohoku University Graduate School of Medicine. Foxp3-RFP reporter mice were obtained from Jackson Laboratory (Bar Harbor, ME) (49). *Cars2* ^+/-^ mice are previously described (11). Since *Cars2* ^-/-^ are embryonically lethal, we used *Cars2* ^+/-^ mice in this study. *Cars2* ^+/-^ Foxp3-RFP reporter mice were obtained by crossing Foxp3-RFP reporter with *Cars2* ^+/-^ mice. All mice were maintained in specific pathogen-free environment in Tohoku University Graduate School of Medicine. The care and handling of the animals used in our study were in accordance with the animal study protocols approved by the Institutional Committee for the Use and Care of Laboratory Animals of Tohoku University (2019MdA-204-04).

### Synthesis of oxidized glutathione trisulfide

GSSSG was synthesized according to our previous reports (11, 32, 50). In brief, 20 mM GSH was reacted with 20 mM NaHS in 20 mM Tris-HCl buffer (pH 7.4) containing 20 mM iodine at room temperature for 15 min. For purification of GSSSG, the reaction mixture was subjected to high-performance LC Prominence (Shimadzu Corporation, Kyoto, Japan) with a reversed-phase column YMC-Triart C18 column, 50×2.0 mm inner diameter (YMC, Kyoto, Japan), under the following elution conditions: mobile phase A (0.1% formic acid) with a linear gradient of mobile phase B (0.1% formic acid in methanol) from 5 to 90% for 15 min at a flow rate of 0.2 ml/min at 40 °C. Eluted GSSSG was dried in vacuo.

### Adoptive transfer

To sort for naïve CD4^+^ T lymphocytes, CD4^+^ cells were enriched from whole splenocytes using CD4 Microbeads (Miltenyi Biotec, Bergisch Gladbach, Germany). Naïve cells (CD3^+^ CD4^+^ CD25^-^ CD44^lo^ CD62L^hi^ or CD3^+^ CD4^+^ Foxp3-RFP^-^ CD44^lo^ CD62L^hi^) were then purified by FACS Aria II (BD Biosciences, San Jose, CA) (**Supplementary Figure S12A**). To obtain Tregs, CD3^+^ CD4^+^ Foxp3-RFP^+^ cells were sorted out. For examination of homeostatic proliferation, purified naïve CD4^+^ T cells were labeled with CFSE (Thermo Fisher Scientific, Waltham, MA) and injected intravenously into sublethally irradiated (5.5Gy) CD45.1^+^ WT recipient mice (1 x 10^6^ cells per recipient) as previously described (18). To induce colitis, WT or *Cars2* ^+/-^ naïve CD4^+^ T cells were injected into *Rag2* ^-/-^ mice (3 x 10^5^ cells per recipient). In **Supplementary Figure S9**, *Cars2* ^+/+^ naïve CD4^+^ T cells were co-transferred with Tregs (1 x 10^5^ cells per mouse) derived from *Cars2* ^+/+^ or *Cars2* ^+/-^ Foxp3-RFP reporter mice. For *in vivo* GSSSG treatment, *Rag2* ^-/-^ recipient mice were daily injected intraperitoneally with GSSSG (400 nmol per mouse) starting on the same day of naïve CD4^+^ T cell transfer.

### Histological assessment of intestinal inflammation

Three to four weeks after naive cell transfer, colons of *Rag2* ^-/-^ hosts were fixed in 10% formalin. Paraffin-embedded samples were then cut into 5 μm sections and stained with hematoxylin and eosin (H&E) or CD4 (EPR19514) monoclonal antibody (mAb) (Abcam, Trumpington, UK). Images were acquired using BZ-X810 (Keyence, Osaka, Japan). The histological score was measured as previously described (51).

### Lymphocyte isolation

Single-cell suspensions from the thymus, spleen, and mLNs were prepared and red blood cells lyzed in ACK buffer. Colonic lamina propria cells were isolated using Lamina Propria Dissociation Kit (Miltenyi Biotec) according to the manufacturer’s instructions. Lymphocytes were then separated using Percoll (GE Healthcare, Chicago, IL).

### Real-time qPCR

For the measurement of *Cars2* mRNA in cultured CD4^+^ T cells, naïve CD4^+^ T cells stimulated with plate-coated CD3 (1 µg/ml) (Biolegend, San Diego, CA) were collected. For the measurement of *Trp53* mRNA in cultured CD4^+^ T cells, naïve CD4^+^ T cells stimulated with plate-coated CD3 (1 µg/ml) and soluble CD28 (1 μg/ml) (both from Biolegend) were collected. For detection of *Trp53* mRNA in CD4^+^ T cells *in vivo*, colonic CD4^+^ T cells were sorted out. Total RNA was extracted using the RNeasy Mini Kit (Qiagen, Hilden, Germany) and reverse-transcribed with the High Capacity cDNA Reverses Transcriptase Kit (Thermo Fisher Scientific). Real-time PCR was performed using FastStart Universal SYBR qPCR Mix (TOYOBO, Osaka, Japan). qPCR analysis was carried out using the ABI 7500 Real-time PCR System (Thermo Fisher Scientific). Relative gene expression was calculated by the ΔCt method and normalized to the amount of Actin-beta (*Actb*). The following primer sets were used: *Trp53*: 5’-ACGCTTCTCCGAAGACTGG-3’ and 5’-AGGGAGCTCGAGGCTGATA-3’; *Cars2*: 5’-CAGGTGCATAACAGCCTCACT-3’ and 5’-CCACAGCTATACCAGGAGACTG-3’; *Actb*: 5’-GAAGATCAAGATCATTGCTCCT-3’ and 5’-TGGAAGGTGGACAGTGAG-3’.

### *In vitro* culture of murine naive CD4^+^ T cells

In **Figure 4** and **Supplementary Figure S8**, naïve CD4^+^ T cells (5 x 10^4^ cells per well) isolated by Naïve CD4^+^ T cell isolation Kit (Miltenyi Biotec) were stimulated with antibodies against plate-coated CD3 (1 μg/ml) and soluble CD28 (1 μg/ml) (both from Biolegend) in RPMI complete medium supplemented with 50 μM β-mercaptoethanol for 2 to 3 days. For Th1 condition, recombinant mouse (rm) IL-12 (20 ng/ml; ThermoFisher) and anti-IL-4 antibody (11B11) (5 μg/ml; Biolegend) were added. For Th2 condition, rmIL-4 (10 ng/ml; Biolegend) and anti-IFN-γ antibody (XMG1.2) (10 μg/ml; Biolegend) were added. For Th17 condition, recombinant human (rh) IL-6 (30 ng/ml; ThermoFisher), rhIL-6R (66 ng/ml; ThermoFisher), anti-IFN-γ antibody (10 μg/ml; Biolegend) and anti-IL-4 antibody (5 μg/ml; Biolegend) were added. For Treg condition, rhTGF-β (0.5 ng/ml; ThermoFisher), anti-IFN-γ antibody (10 μg/ml; Biolegend) and anti-IL-4 antibody (5 μg/ml; Biolegend) were added. In **Figures 5**, FACS-sorted *Cars2* ^+/-^ naïve CD4^+^ T cells (5 x 10^4^ cells per well) were stimulated with antibodies against plate-coated CD3 (1 μg/ml) and soluble CD28 (1 μg/ml) (both from Biolegend) in RPMI complete media supplemented with 50 μM β-mercaptoethanol for 3 days in the presence or absence of GSSSG (1 μM).

### Flow cytometric analysis

Cells were incubated with CD16/32 mAb (for mice) or Fc receptor blocking solution (for humans; Biolegend) and stained with combinations of the following mAbs for 20 min on ice: CD3 (145-2C11), CD62L (MEL-14) (Thermo Fischer Scientific), CD4 (RM4-5), CD44 (IM7) (BD Biosciences), CD8α (53-6.7), CD25 (PC61), CD45.1 (A20), CD45.2 (104), CD69 (H1.2F3) (Biolegend) for mouse samples, and CD3 (SK7), CD4 (SK3), CD25 (M-A251), CD45RA (HI100), and CD45RO (UCHL1) (BD Biosciences) for human samples. Dead cells were removed using LIVE/DEAD fixable dead cell stain kit (Thermo Fisher Scientific). To detect intracellular antigens, cells were fixed and permeabilized using Foxp3/Transcription Factor Staining Buffer Set (Thermo Fisher Scientific) for 30 min on ice after cell surface staining, followed by staining with mAbs against Foxp3 (FJK-16s), Ki67 (SolA15) (Thermo Fischer Scientific), IFN-γ (XMG1.2), IL-13 (eBio13A) and/or IL-17A (TC11-18H101) (Biolegend) for 30 min at room temperature. For cell cycle analysis, cells were incubated with 2 μg/ml Hoechst 33342 (Thermo Fischer Scientific) for 15 min after intracellular staining as previously described (52). Flow cytometry was performed using LSR Fortessa and the data analyzed with FlowJo software (both BD Biosciences). We counted the number of cells in each cell subset during flow cytometry analysis. The gating strategy is detailed in **Supplementary Figure S12B**.

### Analysis of human transcriptomic data

Public transcriptomic datasets on colonic T lymphocytes obtained from CD patients and controls were downloaded from Gene Expression Omnibus website (http://www.ncbi.nlm.nih.gov/geo/) under the accession number GSE157477 (25) and Broad DUOS (https://duos.broadinstitute.org) under the accession number DUOS-000146 CD_Atlas_2021_GIDER; DUOS-000145 CD_Atlas_2021_PRISM (26) and re-analyzed using R with the package Seurat version 4.0 (http://satijalab.org/seurat/) (53). Analyzed samples were age matched (> 45 years old) and genes detected in less than five cells were excluded. Low-quality cells or empty droplets defined as those with expression of less than 3,000 genes were filtered out.

### Isolation and culture of human naïve CD4^+^ T cells

Whole blood samples were collected from healthy donors under the approval of Institutional Review Boards of Tohoku University Graduate School of Medicine (2021-1-1249). Peripheral blood mononuclear cells were prepared using Vacutainer (BD Biosciences) according to the manufacture’s protocols and purified for naïve CD4^+^ T cells (CD3^+^ CD4^+^ CD25^-^ CD45RA^+^ CD45RO^-^) by using FACS Aria II (BD Biosciences) (**Supplementary Figure S12C**). Cells were then seeded onto a 96 well round bottom plate (5 x 10^4^ cells/well) and stimulated with Dynabeads™ Human T-Activator CD3/CD28 (Thermo Fisher Scientific) in RPMI complete media in the presence or absence of GSSSG (1 μM) for 48 hours.

### Statistical analysis

For re-analysis of human single cell RNA sequencing data, we conducted a Wilcoxson Rank Sum test to establish statistical significance. In human T cell culture experiments, a paired Student’s t-test was performed. In all other instances, an unpaired t-test was applied. *p* values <0.05 were considered to be statistically significant.

## Data Availability

The original contributions presented in the study are included in the article/**Supplementary Material**. Further inquiries can be directed to the corresponding authors.

## References

[1] DeFilippis EM Longman R Harbus M Dannenberg K Scherl EJ. Crohn’s disease: evolution, epigenetics, and the emerging role of microbiome-targeted therapies. Curr Gastroenterol Rep. (2016) 18:13. doi: 10.1007/s11894-016-0487-z 26908281

[2] Imam T Park S Kaplan MH Olson MR. Effector T helper cell subsets in inflammatory bowel diseases. Front Immunol. (2018) 9:1212. doi: 10.3389/fimmu.2018.01212 29910812 PMC5992276

[3] Ben-Horin S Chowers Y. Tailoring anti-TNF therapy in IBD: drug levels and disease activity. Nat Rev Gastroenterol Hepatol. (2014) 11:243–55. doi: 10.1038/nrgastro.2013.253 24393836

[4] Qiu Y Chen BL Mao R Zhang SH He Y Zeng ZR. Systematic review with meta-analysis: loss of response and requirement of anti-TNFα dose intensification in Crohn’s disease. J Gastroenterol. (2017) 52:535–54. doi: 10.1007/s00535-017-1324-3 28275925

[5] Abraham C Dulai PS Vermeire S Sandborn WJ. Lessons learned from trials targeting cytokine pathways in patients with inflammatory bowel diseases. Gastroenterology. (2017) 152:374–388.e374. doi: 10.1053/j.gastro.2016.10.018 27780712 PMC5287922

[6] Chapman NM Boothby MR Chi H. Metabolic coordination of T cell quiescence and activation. Nat Rev Immunol. (2020) 20:55–70. doi: 10.1038/s41577-019-0203-y 31406325

[7] MacIver NJ Michalek RD Rathmell JC. Metabolic regulation of T lymphocytes. Annu Rev Immunol. (2013) 31:259–83. doi: 10.1146/annurev-immunol-032712-095956 PMC3606674 23298210

[8] Wang R Dillon CP Shi LZ Milasta S Carter R Finkelstein D. The transcription factor Myc controls metabolic reprogramming upon T lymphocyte activation. Immunity. (2011) 35:871–82. doi: 10.1016/j.immuni.2011.09.021 PMC3248798 22195744

[9] Endo Y Onodera A Obata-Ninomiya K Koyama-Nasu R Asou HK Ito T. ACC1 determines memory potential of individual CD4(+) T cells by regulating *de novo* fatty acid biosynthesis. Nat Metab. (2019) 1:261–75. doi: 10.1038/s42255-018-0025-4 32694782

[10] Zhang D Jin W Wu R Li J Park SA Tu E. High glucose intake exacerbates autoimmunity through reactive-oxygen-species-mediated TGF-β Cytokine activation. Immunity. (2019) 51:671–681.e675. doi: 10.1016/j.immuni.2019.08.001 31451397 PMC9811990

[11] Akaike T Ida T Wei FY Nishida M Kumagai Y Alam MM. Cysteinyl-tRNA synthetase governs cysteine polysulfidation and mitochondrial bioenergetics. Nat Commun. (2017) 8:1177. doi: 10.1038/s41467-017-01311-y 29079736 PMC5660078

[12] Khan S Fujii S Matsunaga T Nishimura A Ono K Ida T. Reactive persulfides from salmonella typhimurium downregulate autophagy-mediated innate immunity in macrophages by inhibiting electrophilic signaling. Cell Chem Biol. (2018) 25:1403–1413.e1404. doi: 10.1016/j.chembiol.2018.08.007 30197193

[13] Zhang T Ono K Tsutsuki H Ihara H Islam W Akaike T. Enhanced cellular polysulfides negatively regulate TLR4 signaling and mitigate lethal endotoxin shock. Cell Chem Biol. (2019) 26:686–698.e684. doi: 10.1016/j.chembiol.2019.02.003 30853417

[14] Matsunaga T Sano H Takita K Morita M Yamanaka S Ichikawa T. Supersulphides provide airway protection in viral and chronic lung diseases. Nat Commun. (2023) 14:4476. doi: 10.1038/s41467-023-40182-4 37491435 PMC10368687

[15] Numakura T Sugiura H Akaike T Ida T Fujii S Koarai A. Production of reactive persulfide species in chronic obstructive pulmonary disease. Thorax. (2017) 72:1074–83. doi: 10.1136/thoraxjnl-2016-209359 28724639

[16] Kawabe T Yi J Sprent J. Homeostasis of naive and memory T lymphocytes. Cold Spring Harb Perspect Biol. (2021) 13:a037879. doi: 10.1101/cshperspect.a037879 33753403 PMC8411951

[17] Ernst B Lee DS Chang JM Sprent J Surh CD. The peptide ligands mediating positive selection in the thymus control T cell survival and homeostatic proliferation in the periphery. Immunity. (1999) 11:173–81. doi: 10.1016/s1074-7613(00)80092-8 10485652

[18] Kawabe T Sun SL Fujita T Yamaki S Asao A Takahashi T. Homeostatic proliferation of naive CD4+ T cells in mesenteric lymph nodes generates gut-tropic Th17 cells. J Immunol. (2013) 190:5788–98. doi: 10.4049/jimmunol.1203111 23610141

[19] Min B Yamane H Hu-Li J Paul WE. Spontaneous and homeostatic proliferation of CD4 T cells are regulated by different mechanisms. J Immunol. (2005) 174:6039–44. doi: 10.4049/jimmunol.174.10.6039 15879097

[20] Malmström V Shipton D Singh B Al-Shamkhani A Puklavec MJ Barclay AN. CD134L expression on dendritic cells in the mesenteric lymph nodes drives colitis in T cell-restored SCID mice. J Immunol. (2001) 166:6972–81. doi: 10.4049/jimmunol.166.11.6972 11359859

[21] Takeda I Ine S Killeen N Ndhlovu LC Murata K Satomi S. Distinct roles for the OX40-OX40 ligand interaction in regulatory and nonregulatory T cells. J Immunol. (2004) 172:3580–9. doi: 10.4049/jimmunol.172.6.3580 15004159

[22] Morrissey PJ Charrier K Braddy S Liggitt D Watson JD. CD4+ T cells that express high levels of CD45RB induce wasting disease when transferred into congenic severe combined immunodeficient mice. Disease development is prevented by cotransfer of purified CD4+ T cells. J Exp Med. (1993) 178:237–44. doi: 10.1084/jem.178.1.237 PMC2191069 8100269

[23] Powrie F Leach MW Mauze S Caddle LB Coffman RL. Phenotypically distinct subsets of CD4+ T cells induce or protect from chronic intestinal inflammation in C. B-17 scid mice. Int Immunol. (1993) 5:1461–71. doi: 10.1093/intimm/5.11.1461 7903159

[24] Leppkes M Becker C Ivanov II Hirth S Wirtz S Neufert C. RORgamma-expressing Th17 cells induce murine chronic intestinal inflammation via redundant effects of IL-17A and IL-17F. Gastroenterology. (2009) 136:257–67. doi: 10.1053/j.gastro.2008.10.018 18992745

[25] Jaeger N Gamini R Cella M Schettini JL Bugatti M Zhao S. Single-cell analyses of Crohn’s disease tissues reveal intestinal intraepithelial T cells heterogeneity and altered subset distributions. Nat Commun. (2021) 12:1921. doi: 10.1038/s41467-021-22164-6 33771991 PMC7997960

[26] Kong L Pokatayev V Lefkovith A Carter GT Creasey EA Krishna C. The landscape of immune dysregulation in Crohn’s disease revealed through single-cell transcriptomic profiling in the ileum and colon. Immunity. (2023) 56:444–458.e445. doi: 10.1016/j.immuni.2023.01.002 36720220 PMC9957882

[27] Martin JC Chang C Boschetti G Ungaro R Giri M Grout JA. Single-cell analysis of Crohn’s disease lesions identifies a pathogenic cellular module associated with resistance to anti-TNF therapy. Cell. (2019) 178:1493–1508.e1420. doi: 10.1016/j.cell.2019.08.008 31474370 PMC7060942

[28] Tan SY Kelkar Y Hadjipanayis A Shipstone A Wynn TA Hall JP. Metformin and 2-deoxyglucose collaboratively suppress human CD4(+) T cell effector functions and activation-induced metabolic reprogramming. J Immunol. (2020) 205:957–67. doi: 10.4049/jimmunol.2000137 32641388

[29] Szabo PA Levitin HM Miron M Snyder ME Senda T Yuan J. Single-cell transcriptomics of human T cells reveals tissue and activation signatures in health and disease. Nat Commun. (2019) 10:4706. doi: 10.1038/s41467-019-12464-3 31624246 PMC6797728

[30] Wang X Shen X Chen S Liu H Hong N Zhong H. Reinvestigation of classic T cell subsets and identification of novel cell subpopulations by single-cell RNA sequencing. J Immunol. (2022) 208:396–406. doi: 10.4049/jimmunol.2100581 34911770

[31] Fujii S Sawa T Motohashi H Akaike T. Persulfide synthases that are functionally coupled with translation mediate sulfur respiration in mammalian cells. Br J Pharmacol. (2019) 176:607–15. doi: 10.1111/bph.14356 PMC6346073 29748969

[32] Ida T Sawa T Ihara H Tsuchiya Y Watanabe Y Kumagai Y. Reactive cysteine persulfides and S-polythiolation regulate oxidative stress and redox signaling. Proc Natl Acad Sci U.S.A. (2014) 111:7606–11. doi: 10.1073/pnas.1321232111 PMC4040604 24733942

[33] Morikawa T Kajimura M Nakamura T Hishiki T Nakanishi T Yukutake Y. Hypoxic regulation of the cerebral microcirculation is mediated by a carbon monoxide-sensitive hydrogen sulfide pathway. Proc Natl Acad Sci U.S.A. (2012) 109:1293–8. doi: 10.1073/pnas.1119658109 PMC3268316 22232681

[34] Nakano S Ishii I Shinmura K Tamaki K Hishiki T Akahoshi N. Hyperhomocysteinemia abrogates fasting-induced cardioprotection against ischemia/reperfusion by limiting bioavailability of hydrogen sulfide anions. J Mol Med (Berl). (2015) 93:879–89. doi: 10.1007/s00109-015-1271-5 25740079

[35] Nishida M Sawa T Kitajima N Ono K Inoue H Ihara H. Hydrogen sulfide anion regulates redox signaling via electrophile sulfhydration. Nat Chem Biol. (2012) 8:714–24. doi: 10.1038/nchembio.1018 PMC4123552 22772154

[36] Shirozu K Tokuda K Marutani E Lefer D Wang R Ichinose F. Cystathionine gamma-lyase deficiency protects mice from galactosamine/lipopolysaccharide-induced acute liver failure. Antioxid Redox Signal. (2014) 20:204–16. doi: 10.1089/ars.2013.5354 PMC3887435 23758073

[37] Yadav PK Martinov M Vitvitsky V Seravalli J Wedmann R Filipovic MR. Biosynthesis and reactivity of cysteine persulfides in signaling. J Am Chem Soc. (2016) 138:289–99. doi: 10.1021/jacs.5b10494 PMC4795164 26667407

[38] Zainol Abidin QH Ida T Morita M Matsunaga T Nishimura A Jung M. Synthesis of sulfides and persulfides is not impeded by disruption of three canonical enzymes in sulfur metabolism. Antioxid (Basel). (2023) 12:868. doi: 10.3390/antiox12040868 PMC10135671 37107243

[39] Yang R Qu C Zhou Y Konkel JE Shi S Liu Y. Hydrogen sulfide promotes Tet1- and Tet2-mediated Foxp3 demethylation to drive regulatory T cell differentiation and maintain immune homeostasis. Immunity. (2015) 43:251–63. doi: 10.1016/j.immuni.2015.07.017 PMC4731232 26275994

[40] Sasaki Y Numakura T Yamada M Sugiura H Matsunaga T Ida T. Glutathione supersulphide regulates T-cell receptor signalling. bioRxiv. (2024) 2024.04.30.591985. doi: 10.1101/2024.04.30.591985

[41] Capuozzo M Santorsola M Bocchetti M Perri F Cascella M Granata V. p53: from fundamental biology to clinical applications in cancer. Biol (Basel). (2022) 11:1325. doi: 10.3390/biology11091325 PMC9495382 36138802

[42] Ho JS Ma W Mao DY Benchimol S. p53-Dependent transcriptional repression of c-myc is required for G1 cell cycle arrest. Mol Cell Biol. (2005) 25:7423–31. doi: 10.1128/mcb.25.17.7423-7431.2005 PMC1190302 16107691

[43] Watanabe M Moon KD Vacchio MS Hathcock KS Hodes RJ. Downmodulation of tumor suppressor p53 by T cell receptor signaling is critical for antigen-specific CD4(+) T cell responses. Immunity. (2014) 40:681–91. doi: 10.1016/j.immuni.2014.04.006 PMC4073799 24792911

[44] Serpa J. The putative role of gut microbiota in cancer: Cysteine is a pivotal coin. Front Gastroenterol. (2022) 1:966957. doi: 10.3389/fgstr.2022.966957 PMC12952466 41822090

[45] Radeva G Buyse M Hindlet P Beaufils B Walker F Bado A. Regulation of the oligopeptide transporter, PEPT-1, in DSS-induced rat colitis. Dig Dis Sci. (2007) 52:1653–61. doi: 10.1007/s10620-006-9667-2 17372819

[46] Wojtal KA Eloranta JJ Hruz P Gutmann H Drewe J Staumann A. Changes in mRNA expression levels of solute carrier transporters in inflammatory bowel disease patients. Drug Metab Dispos. (2009) 37:1871–7. doi: 10.1124/dmd.109.027367 19487253

[47] Merlin D Si-Tahar M Sitaraman SV Eastburn K Williams I Liu X. Colonic epithelial hPepT1 expression occurs in inflammatory bowel disease: transport of bacterial peptides influences expression of MHC class 1 molecules. Gastroenterology. (2001) 120:1666–79. doi: 10.1053/gast.2001.24845 11375948

[48] Uchiyama J Akiyama M Hase K Kumagai Y Kim YG. Gut microbiota reinforce host antioxidant capacity via the generation of reactive sulfur species. Cell Rep. (2022) 38:110479. doi: 10.1016/j.celrep.2022.110479 35263581

[49] Wan YY Flavell RA. Identifying Foxp3-expressing suppressor T cells with a bicistronic reporter. Proc Natl Acad Sci U.S.A. (2005) 102:5126–31. doi: 10.1073/pnas.0501701102 PMC556008 15795373

[50] Takata T Jung M Matsunaga T Ida T Morita M Motohashi H. Methods in sulfide and persulfide research. Nitric Oxide. (2021) 116:47–64. doi: 10.1016/j.niox.2021.09.002 34534626 PMC8486624

[51] Biswas A Shouval DS Griffith A Goettel JA Field M Kang YH. WASP-mediated regulation of anti-inflammatory macrophages is IL-10 dependent and is critical for intestinal homeostasis. Nat Commun. (2018) 9:1779. doi: 10.1038/s41467-018-03670-6 29725003 PMC5934380

[52] Muñoz-Ruiz M Pujol-Autonell I Rhys H Long HM Greco M Peakman M. Tracking immunodynamics by identification of S-G(2)/M-phase T cells in human peripheral blood. J Autoimmun. (2020) 112:102466. doi: 10.1016/j.jaut.2020.102466 32414606 PMC7527781

[53] Stuart T Butler A Hoffman P Hafemeister C Papalexi E Mauck WM 3rd. Comprehensive integration of single-cell data. Cell. (2019) 177:1888–1902.e1821. doi: 10.1016/j.cell.2019.05.031 31178118 PMC6687398

