# Supplementary material for: Supersulfide controls intestinal inflammation by suppressing CD4+ T cell proliferation
Source: Front Immunol. 2025 Apr 15;16:1506580. doi: 10.3389/fimmu.2025.1506580 (PMC12037617; doi:10.3389/fimmu.2025.1506580)
Supplement: Supplementary file 1 [file DataSheet1.pdf]

**Supplementary Figures**

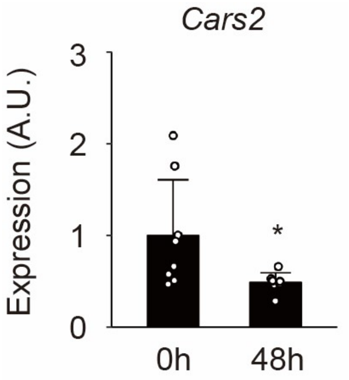


**Supplementary Figure 1. Downregulation of *Cars2* in CD4$^+$ T cells after TCR stimulation.**

WT naïve CD4$^+$ T cells were stimulated with CD3 for 48 hours. A bar graph displays the relative expression of *Cars2* in CD4$^+$ T cells (n = 8). Data shown are pooled from two independent experiments and expressed as mean ± standard deviation. $*p < 0.05$.

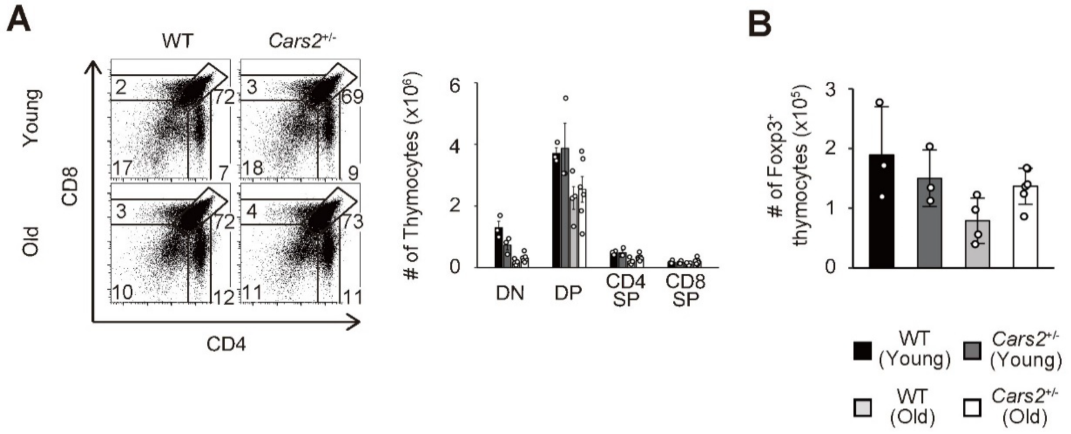


**Supplementary Figure 2. Normal development of thymic T cells in *Cars2*$^{+/-}$ mice.**
(**A**) Dot plots depicting expression of CD4 and CD8 in thymocytes from the indicated animals, together with a bar graph showing the number of DN, DP, and CD4 and CD8 SP cells (n = 3 to 6). (**B**) Bar graph indicating the absolute number of Foxp3$^{+}$ cells in the thymus (n = 3 to 6). Data shown are pooled from two independent experiments and expressed as mean ± standard deviation.

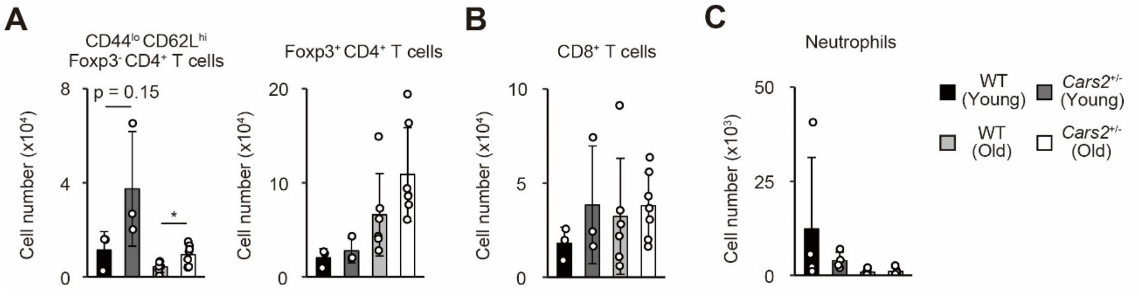


**Supplementary Figure 3.** ***Cars2*****-deficiency and ageing do not affect the infiltration of various immune cells, except for naïve CD4$^{+}$ T cells in the colon.**

(**A - C**) Bar graphs show the total number of (A) naive CD4$^{+}$ T cells and Tregs, (B) CD8$^{+}$ T cells and (C) neutrophils (CD11b$^{+}$ I-A/I-E$^{-}$ Ly6G$^{+}$) (n = 3 to 7). Data shown are pooled from (C) two to (A, B) three independent experiments and expressed as mean ± standard deviation. *$p < 0.05$.

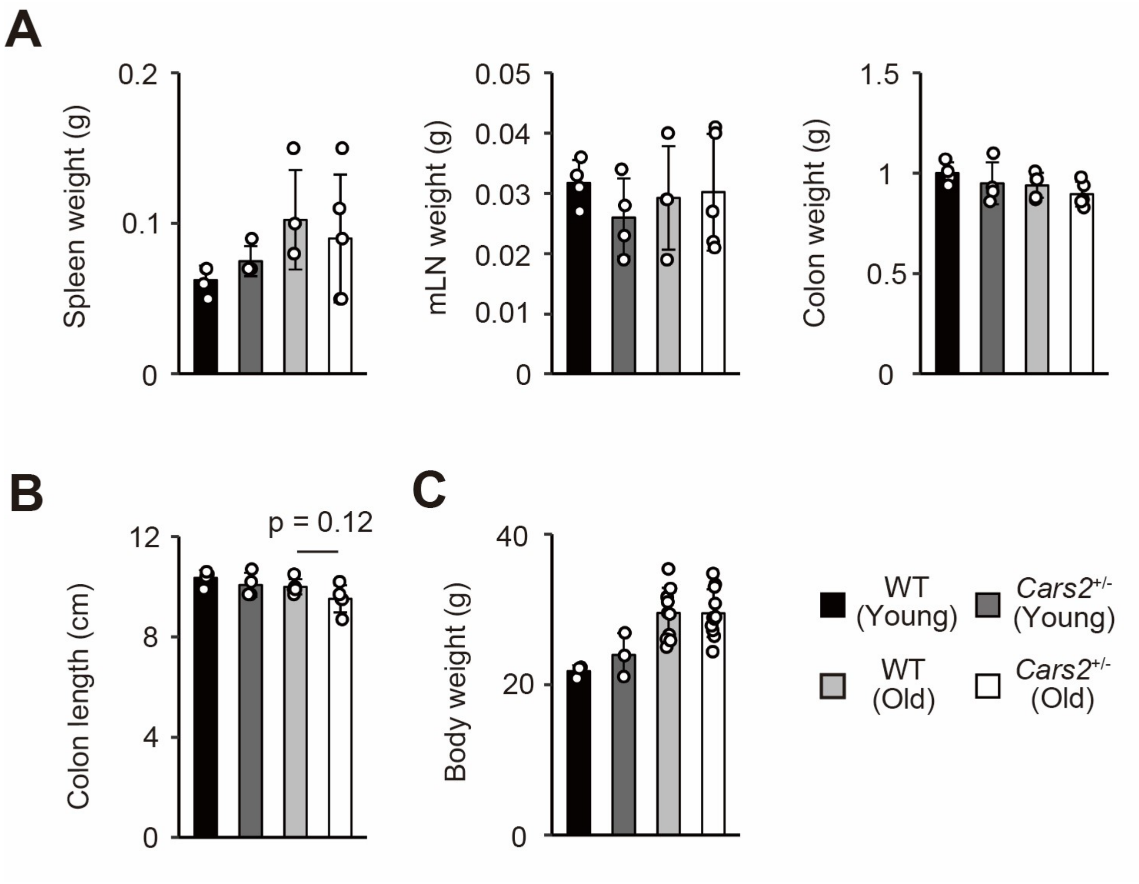


**Supplementary Figure 4. Organs, body weight and colon length of *Cars2*$^{+/-}$ mice.** (**A**) Bar graphs showing the weight of spleen, mLN and colon in the indicated groups (n = 4 to 6) (**B**) Bar graph showing the colon length (n = 4 to 6). (**C**) Bar graph showing body weight (n = 4 to 11). Data shown are pooled from two independent experiments and expressed as mean ± standard deviation.

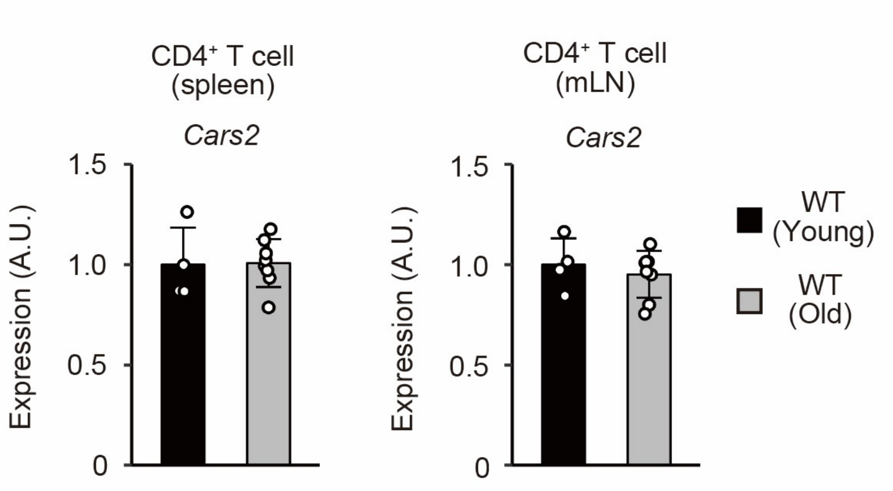


**Supplementary Figure 5. *Cars2* expression in CD4$^+$ T cells extracted from spleen and mLN between WT young and old.**

Bar graphs showing the relative expression level of *Cars2* in CD4$^+$ T cells isolated from the indicated organs (n = 4 to 8). Data shown are pooled from two independent experiments and expressed as mean ± standard deviation.

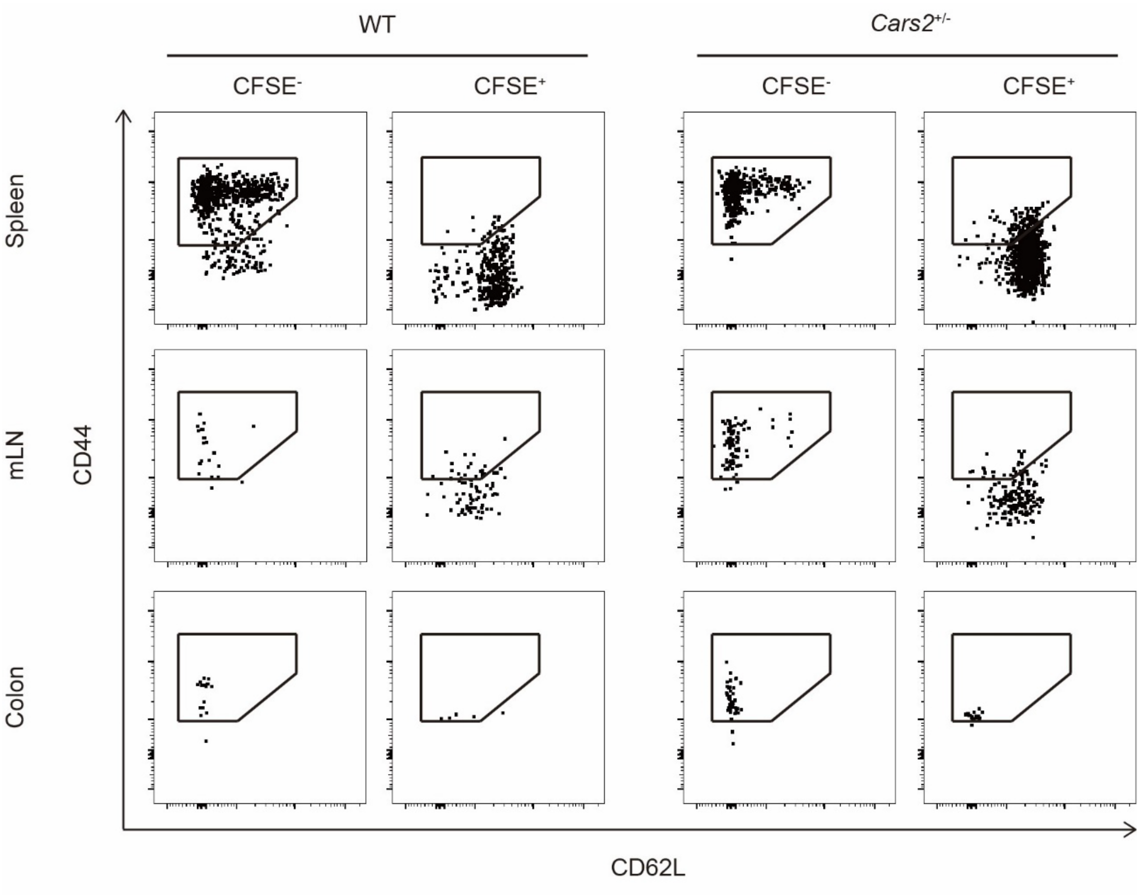


**Supplementary Figure 6. CFSE-negative donor cells are CD44$^{hi}$ CD62L$^{lo}$ effector /memory CD4$^{+}$ T cells.**

The representative dot plots display the expression levels of CD44 and CD62L in CFSE-negative or CFSE-positive donor cells accumulating in the indicated organs.

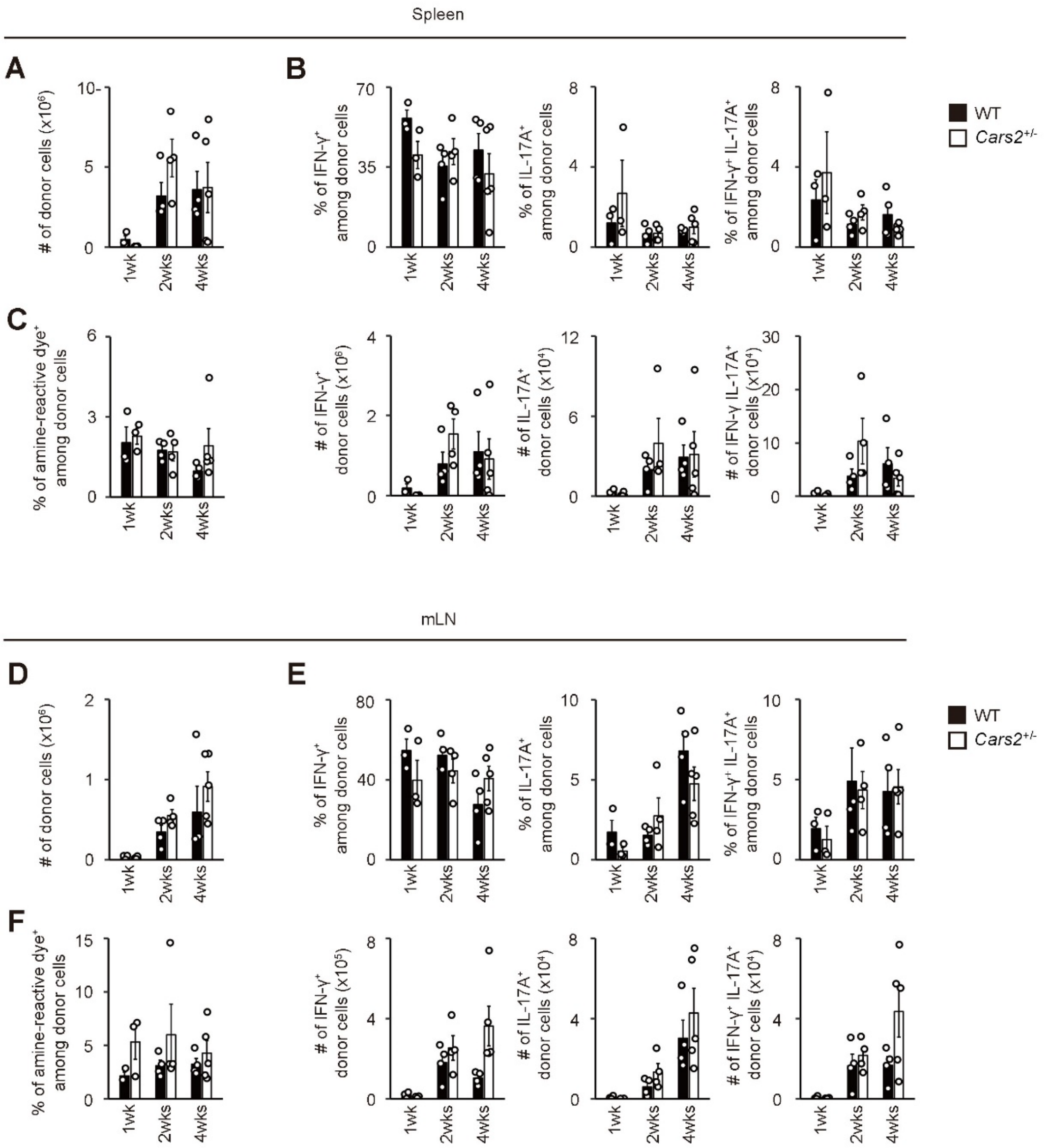


**Supplementary Figure 7. CARS2/CPERS does not significantly affect the accumulation of CD4$^{+}$ T lymphocytes in the spleen or mLNs under inflammatory conditions.**

Colitis was induced in *Rag2*$^{-/-}$ mice as described in Fig. 3 and donor cells were analyzed several weeks later. (**A**) Absolute number of donor cells in the spleen at different time points (n = 3 to 5). (**B**) Frequency and absolute number of cytokine-producing cells among donor cells from the spleen (n = 3 to 5). (**C**) Frequency of amine-reactive dye$^{+}$ (dead) cells among splenic donor cells (n = 3 to 5). (**D**) Absolute number of donor cells in mLNs (n = 3 to 5). (**E**) Frequency and absolute number of cytokine-producing cells among donor cells in mLNs (n = 3 to 5). (**F**) Frequency of amine-reactive dye$^{+}$ (dead) cells among

donor cells in mLNs (n = 3 to 5). Data are pooled from two independent experiments and presented as mean ± standard deviation.

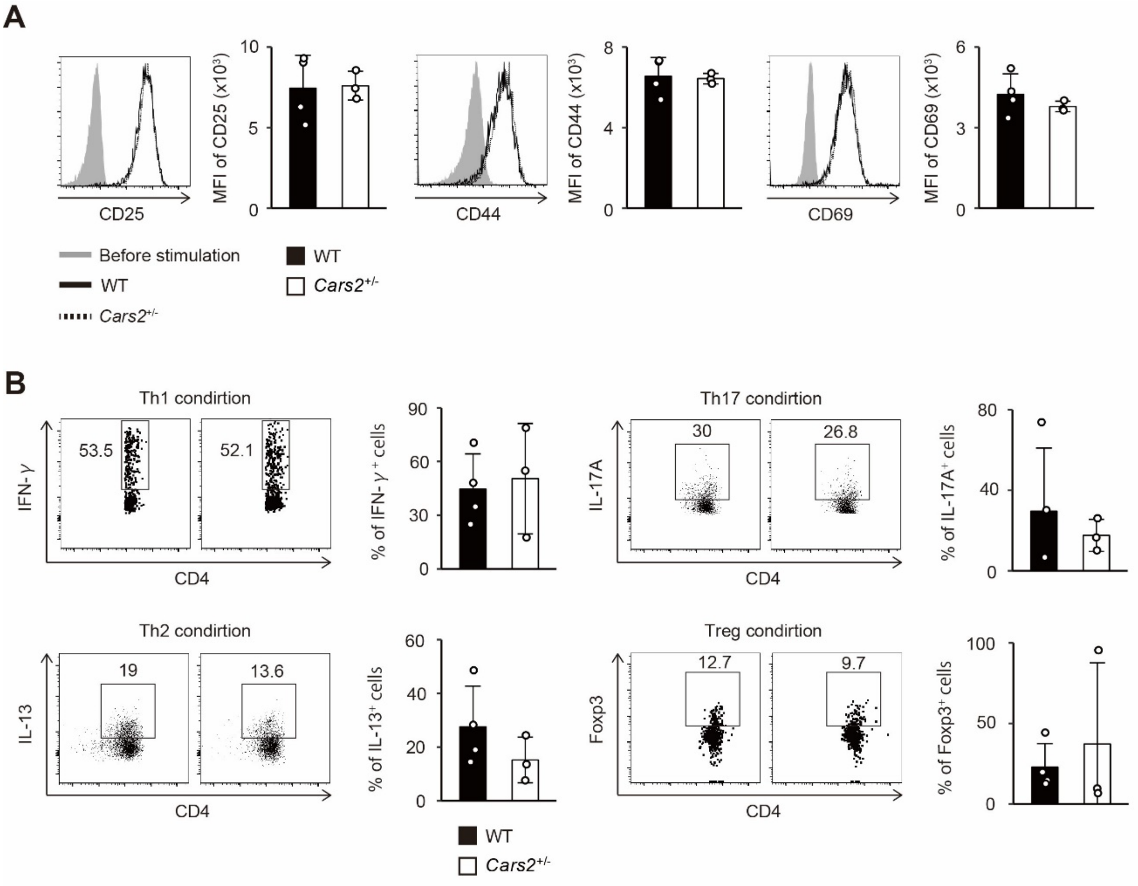


**Supplementary Figure 8. CARS2/CPERS is dispensable for $CD4^+$ T cell activation and differentiation.**

(**A**) Naïve $CD4^+$ T cells were stimulated with CD3 and CD28, and the expression level of activation markers was analyzed 2 days later. Representative histograms show the expression of CD25, CD44, and CD69, while bar graphs indicate the mean fluorescence intensity (MFI) of each marker (n = 3 to 4). (**B**) Naïve $CD4^+$ T cells were cultured in Th1, Th2, Th17, or Treg polarizing conditions. The representative dot plots show the expression level of IFN-γ (Th1), IL-13 (Th2), IL-17A (Th17) and Foxp3 (Treg) in $CD4^+$ T cells after 3 days of culture while the bar graphs show the frequency of polarized T cells (n = 3 to 4). Data shown are representative of two independent experiments and presented as mean ± standard deviation.

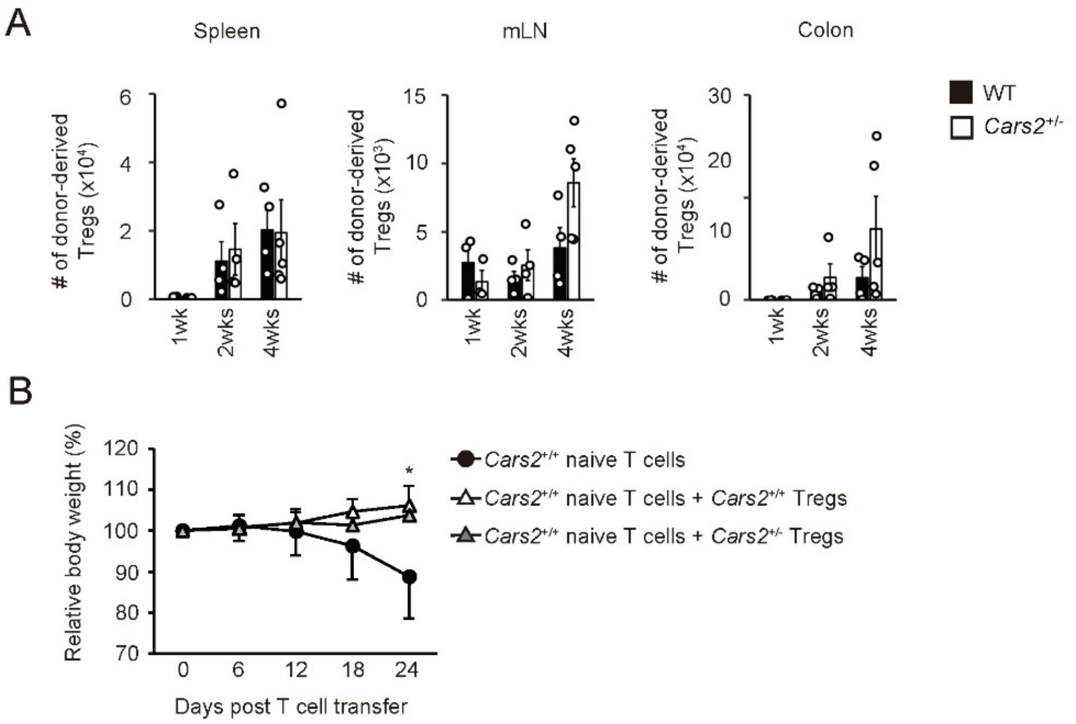


**Supplementary Figure 9. Impairment of CARS2/CPERS in CD4$^+$ T cells does not significantly affect Treg differentiation or suppressive function in *Rag2$^{-/-}$* mice.**

(**A**) Colitis was induced by transfer of naïve CD4$^+$ T lymphocytes into *Rag2$^{-/-}$* mice as described in Fig. 3. Bar graphs indicate the number of newly generated Tregs among the donor cells accumulating in the indicated organs at different time points (n = 3 to 5). (**B**) *Rag2$^{-/-}$* mice received *Cars2$^{+/+}$* Foxp3$^-$ naïve CD4$^+$ T cells alone or together with *Cars2$^{+/+}$* or *Cars2$^{+/-}$* Foxp3$^+$ Tregs. Graph shows the relative body weight of the host animals from each group (n = 4 to 7). Data are pooled from two independent experiments and expressed as mean ± standard deviation. *$p < 0.05$.

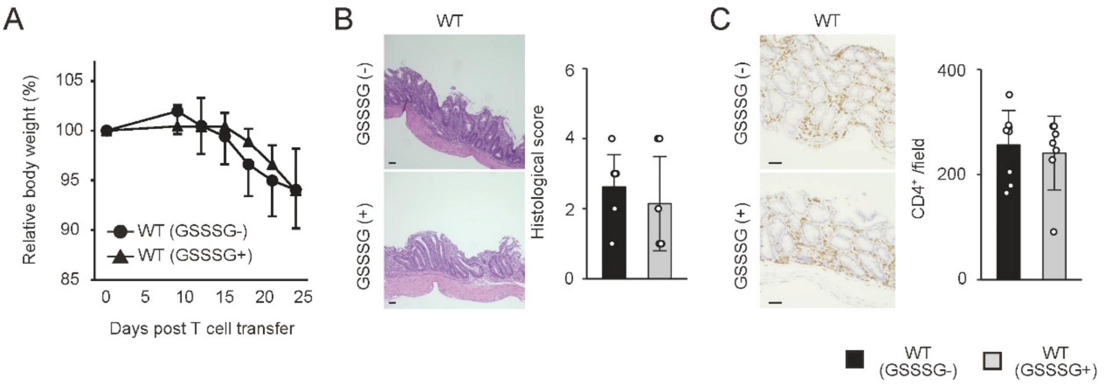


**Supplementary Figure 10. GSSSG treatment show no therapeutic effect in *Rag2*$^{-/-}$ mice transferred with WT naïve CD4$^{+}$ T cells**

WT naïve CD4$^{+}$ T cells had been transferred to *Rag2*$^{-/-}$ hosts that were subsequently treated with PBS or GSSSG. (**A**) Relative body weight of *Rag2*$^{-/-}$ recipient mice from each group at different time points (n = 7 to 8). (**B** and **C**) Representative microscopic images of colon sections stained with (B) H&E and (C) CD4 mAbs along with bar graphs showing (B) histologic scores and (C) quantification of CD4-positive cells (n = 7 to 8). Data are pooled from two independent experiments and presented as mean ± standard deviation. Scale bars, 50 µm.

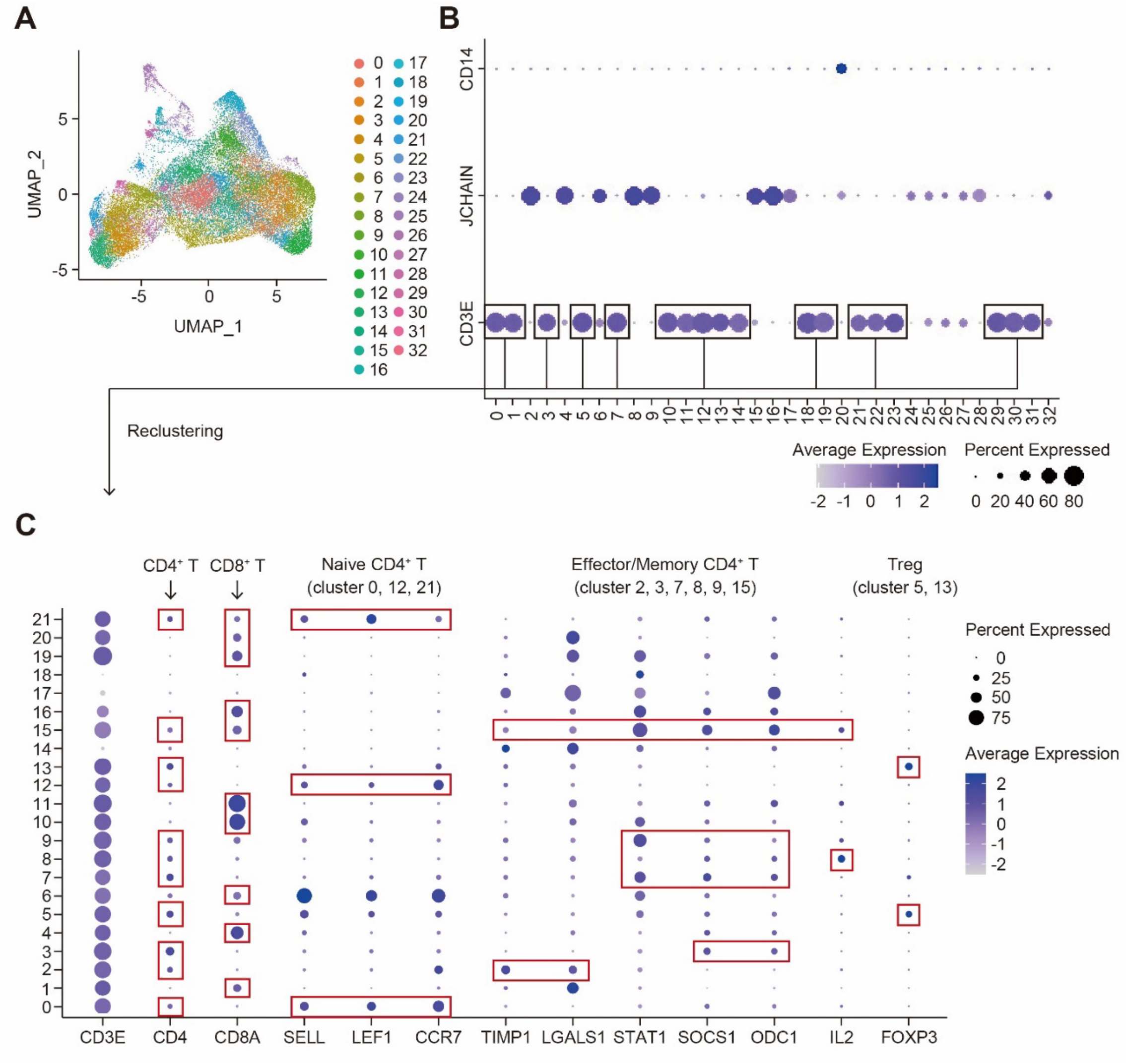


**Supplementary Figure 11. CD3$^+$ and CD4$^+$ T cell subclusters in single-cell RNA sequencing data from human colonic T lymphocytes**

(**A**) Single-cell transcriptomes of colonic cells from CD patients and controls as represented by UMAP. (**B**) Dot plot heat map of T cell, B cell, and myeloid cell markers among total cells. (**C**) Dot plot heat map of representative marker genes for naïve CD4$^+$ T cells, effector CD4$^+$ T cells, Treg and CD8$^+$ T cells among *CD3E*-positive clusters.

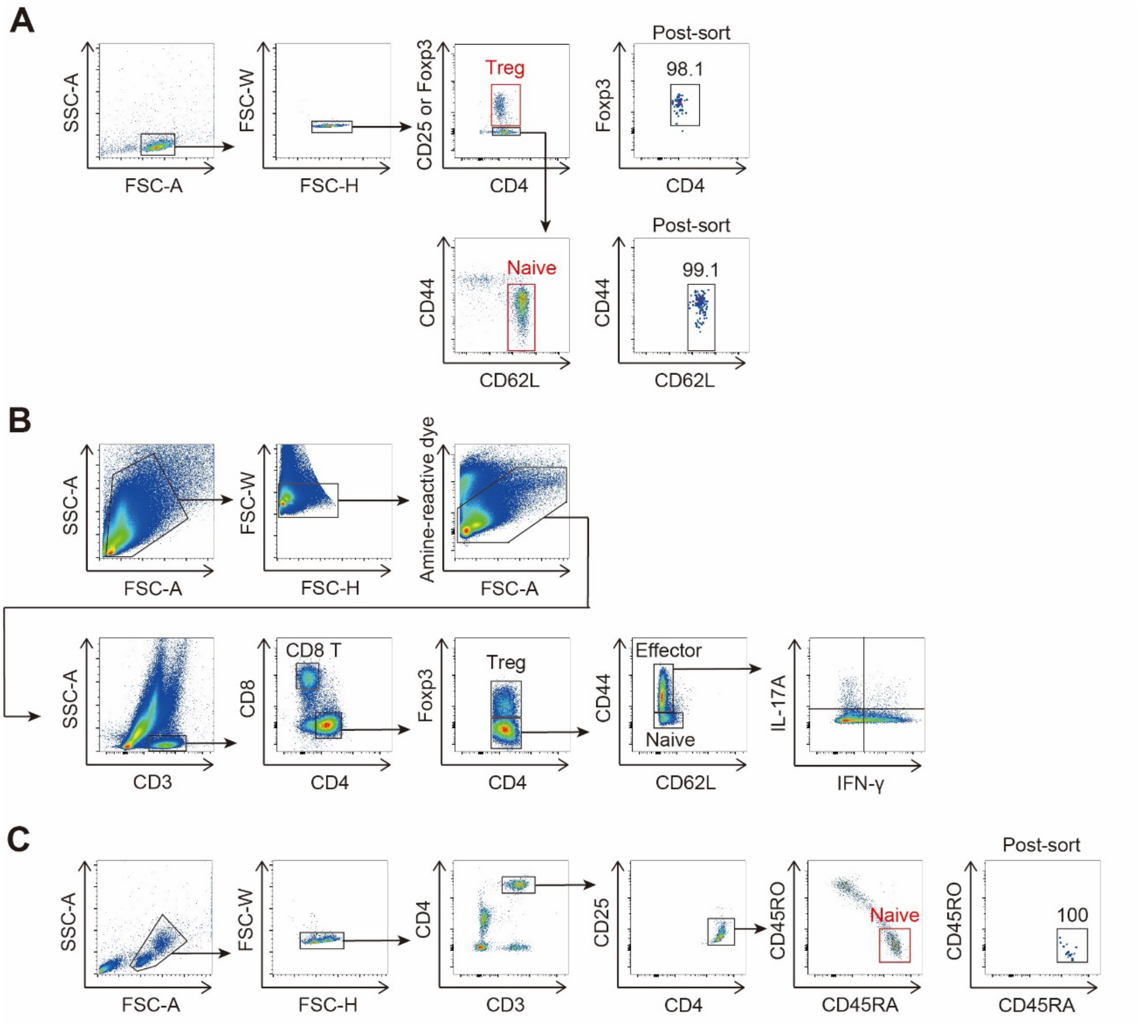


**Supplementary Figure 12. Gating strategy**

(**A**) To sort murine naïve $CD4^+$ T cells and Tregs, total singlet cells were sorted for $CD4^+$ $CD25^-$ (or $Foxp3^-$) $CD44^{lo}$ $CD62L^{hi}$ and $CD4^+$ $Foxp3^+$, respectively. (**B**) To detect mouse T lymphocytes, total cells were gated for live singlet cells. $CD8^+$ T cells, Naïve $CD4^+$ T cells, Effector $CD4^+$ T cells and Tregs were defined as $CD3^+$ $CD8^+$, $CD3^+$ $CD4^+$ $Foxp3^-$ $CD44^{lo}$ $CD62L^{hi}$, $CD3^+$ $CD4^+$ $Foxp3^-$ $CD44^{hi}$ $CD62L^{lo}$, $CD3^+$ $CD4^+$ $Foxp3^+$ populations, respectively. IFN-$\gamma^+$ and IL-$17A^+$ cells are analyzed in effector $CD4^+$ T cells. (**C**) To sort human naïve $CD4^+$ T cells, total singlet cells were sorted for $CD3^+$ $CD4^+$ $CD25^-$ $CD45RA^+$ $CD45RO^-$ cells.
